# Supplementary material for: MARCH5‐dependent NLRP3 ubiquitination is required for mitochondrial NLRP3‐NEK7 complex formation and NLRP3 inflammasome activation
Source: EMBO J. 2023 Aug 14;42(19):e113481. doi: 10.15252/embj.2023113481 (PMC10548170; doi:10.15252/embj.2023113481)
Supplement: Supplementary file 1 — Appendix [file EMBJ-42-e113481-s006.pdf]

MARCH5-dependent NLRP3 ubiquitination is required for mitochondrial NLRP3-NEK7 complex formation and NLRP3 inflammasome activation

Yeon-Ji Park[1,2¶], Niranjan Dodantenna[3¶], Yonghyeon Kim[1,2¶], Tae-Hwan Kim[3], Ho-Soo Lee[1], Young-Suk Yoo[1], June Heo[1,2], Jae-Ho Lee[1], Myung-Hee Kwon[4], Ho Chul Kang[5], Jong-Soo Lee[3]* and Hyeseong Cho[1]*

Appendix

Table of content

1. Appendix Figure S1 page 2-3

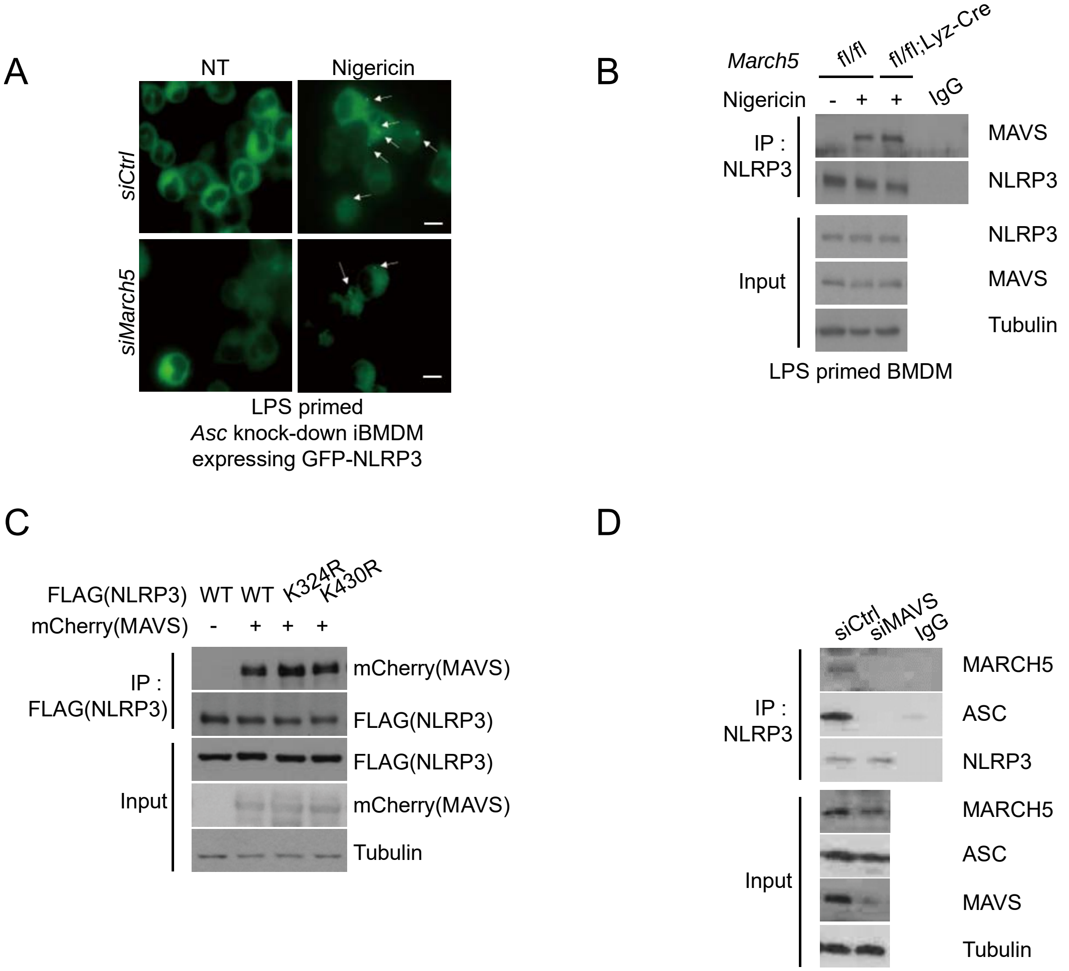

Appendix Figure S1. MAVS is essential for NLRP3-ASC-MARCH5 complex formation.

(A) Fluorescence microscopy images of NLRP3 oligomerization. *Asc* was knocked down in immortalized BMM and cells expressing GFP-NLRP3 as well as *siControl* or *siMarch5*. Cells were stimulated with 200 ng/ml LPS for 4 hr and 15 µM nigericin for 30 min. White arrows indicate NLRP3 oligomerization. Bars, 10 µm.

(B) *March5*$^{fl/fl}$ and *March5*$^{fl/fl;Lyz\text{-}Cre}$ BMDMs were stimulated with 200 ng/ml LPS for 4 hr and 5 mM ATP for 30 min.

(C) HEK293T cells were cotransfected with mCherry-MAVS and WT or mutant NLRP3. Cells were treated with 200 ng/ml LPS

and 15 µM nigericin. Cell lysates were immunoprecipitated with FLAG-M2 beads. The proteins in the precipitate were analyzed by immunoblotting with the indicated antibodies.

(D) THP-1 cells were transfected with siControl and siMAVS. After stimulation with 200 ng/ml LPS for 4 hr and 5 mM ATP for 30 min, cell lysates were immunoprecipitated with NLRP3 antibody. The indicated proteins were detected with the corresponding antibodies.
