# Supplementary material for: MARCH5‐dependent NLRP3 ubiquitination is required for mitochondrial NLRP3‐NEK7 complex formation and NLRP3 inflammasome activation
Source: EMBO J. 2023 Aug 14;42(19):e113481. doi: 10.15252/embj.2023113481 (PMC10548170; doi:10.15252/embj.2023113481)
Supplement: Supplementary file 2 — Expanded View Figures PDF [file EMBJ-42-e113481-s001.pdf]

Expanded View Figures

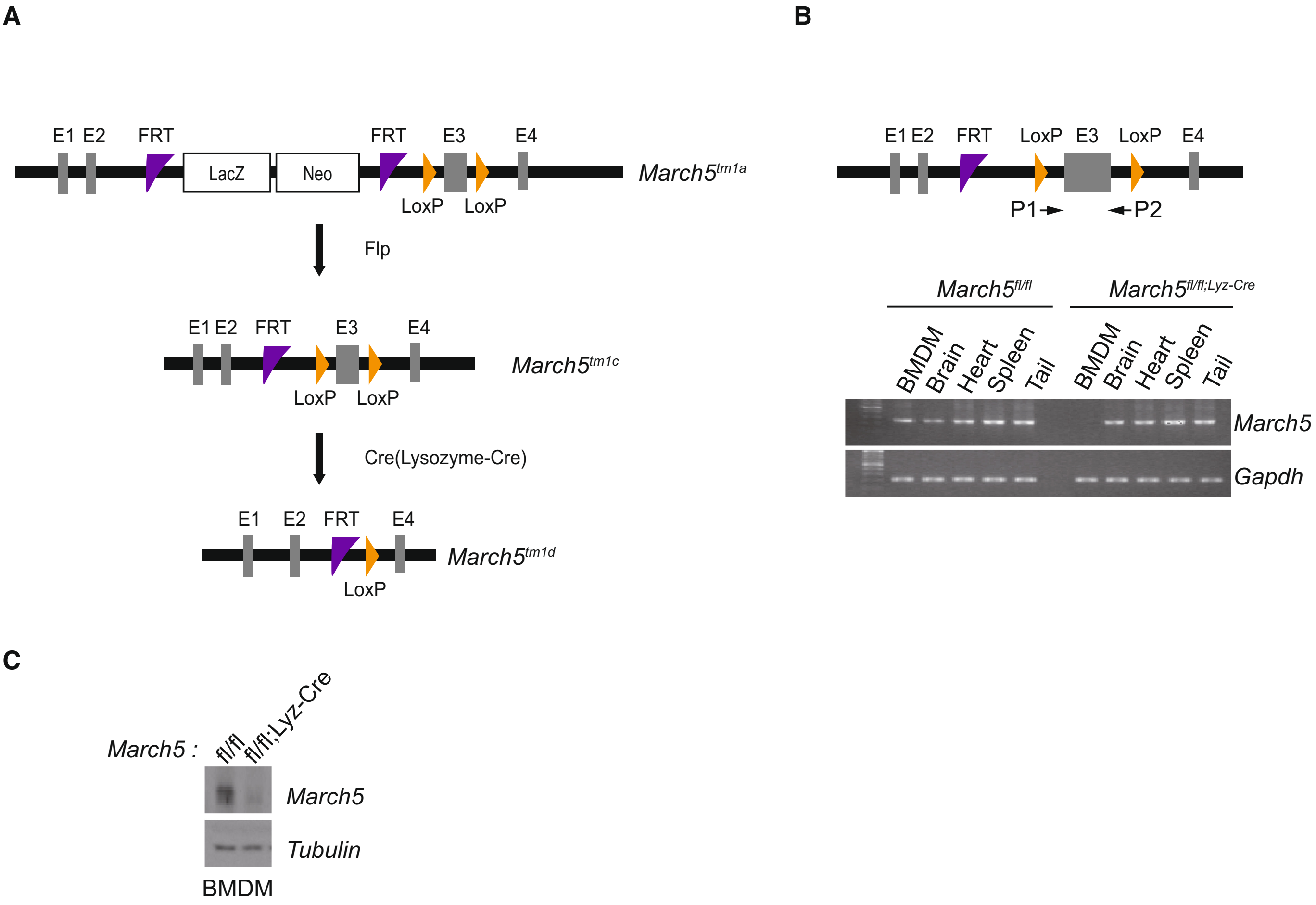

Figure EV1. Generation of *March5*$^{fl/fl}$ and *March5*$^{fl/fl;\ Lyz\text{-}Cre}$ mice.

A A schematic drawing of the *March5* locus and targeting vector. C57BL/6 *March5*^{tm1a} mice harboring the FRT and Lox P sites. generates floxed *March5* mice, Flp recombinase targets FRT sites (*March5*^{tm1c}). For MARCH5 gene deletion, Cre recombinase targets Lox P sites (*March5*^{tm1d}).

B PCR analysis to confirm the deletion of MARCH5 exon 3 by targeted Lysozyme-Cre recombinase. Arrows indicate the sites recognized by the primers. Total mRNA was extracted from the indicated organs from *March5*$^{fl/fl}$ and *March5*$^{fl/fl;Lyz\text{-}Cre}$ mice.

C Bone marrow cells were collected from *March5*$^{fl/fl}$ and *March5*$^{fl/fl;Lyz\text{-}Cre}$ mice and differentiated for 6 days. Western blot analysis of MARCH5 protein expression. Tubulin was used as a loading control.

Source data are available online for this figure.

 © 2023 The Authors

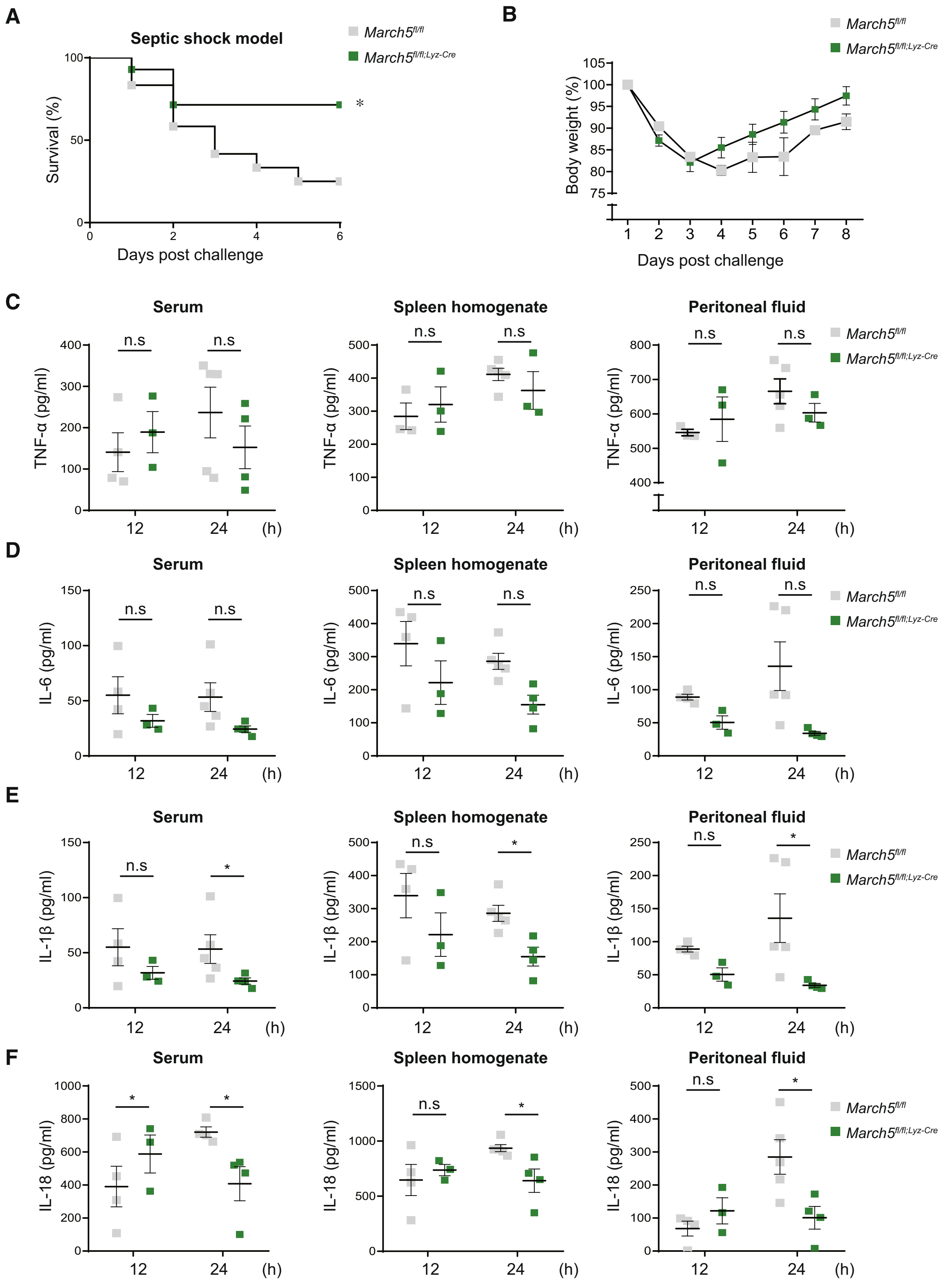
A
Septic shock model
March5fl/fl
March5fl/fl;Lyz-Cre
Survival (%)
Days post challenge
B
Body weight (%)
Days post challenge
C
Serum
Spleen homogenate
Peritoneal fluid
TNF-α (pg/ml)
n.s
(h)
D
IL-6 (pg/ml)
E
IL-1β (pg/ml)
F
IL-18 (pg/ml)

Figure EV2.

Figure EV2. MARCH5 is required for survival and *in vivo* cytokine secretion in response to LPS.

A, B (A) Survival rates (n = 12–14) and (B) variation of body weight of *March5*$^{fl/fl}$ and *March5*$^{fl/fl;Lyz\text{-}Cre}$ (n = 7–9) mice after intraperitoneal injection with 28 mg/kg body weight of LPS.

C–F ELISA of a TNF-α (C), IL-6 (D), IL-1β (E) and IL-18 (F) from serum, spleen homogenate and peritoneal fluid from mice (n = 5), sacrificed 12 and 24 h after LPS injection. Values, *P < 0.05 (two-tailed Student's t-test or Mantel–Cox test.). Data were expressed as the mean ± SEM.

Source data are available online for this figure.

 © 2023 The Authors

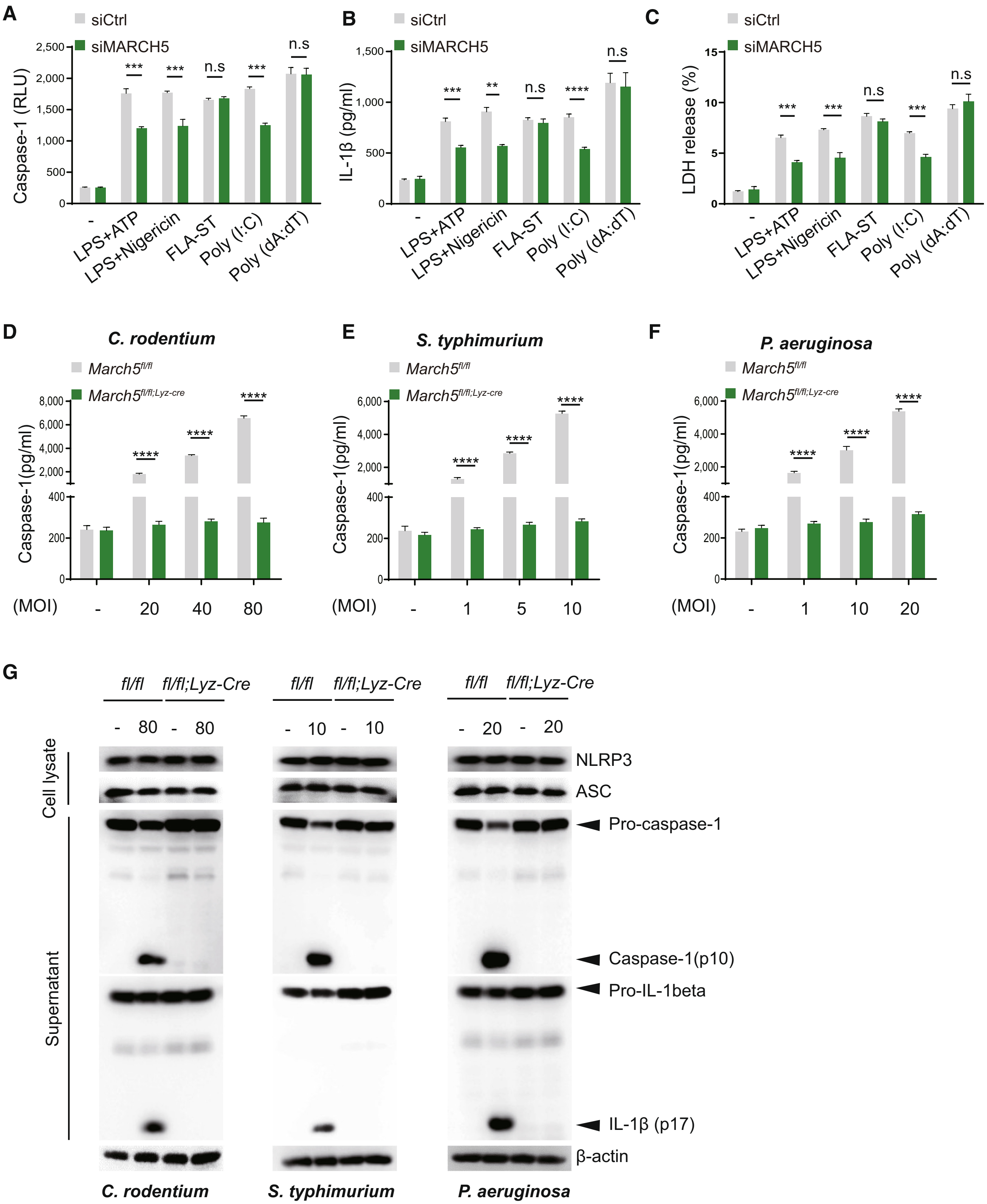

A
siCtrl
siMARCH5
Caspase-1 (RLU)
B
IL-1β (pg/ml)
C
LDH release (%)
-
LPS+ATP
LPS+Nigericin
FLA-ST
Poly (I:C)
Poly (dA:dT)
D
C. rodentium
E
S. typhimurium
F
P. aeruginosa
March5fl/fl
March5fl/fl;Lyz-cre
Caspase-1(pg/ml)
(MOI)
G
fl/fl
fl/fl;Lyz-Cre
Cell lysate
Supernatant
NLRP3
ASC
Pro-caspase-1
Caspase-1(p10)
Pro-IL-1beta
IL-1β (p17)
β-actin

Figure EV3.

Figure EV3. MARCH5 potentiates NLRP3-mediated antimicrobial immunity.

A–C (A) Activated caspase-1, (B) IL-1β, and (C) LDH release were measured in the supernatants of siControl (siCtrl) or siMARCH5 THP-1 cells and were subjected to the indicated stimuli. Independent experiments were repeated at least three times. Values are the mean ± SD. $^{**}P < 0.01$, $^{***}P < 0.001$ (two-tailed student's *t*-test).

D–G *March5*$^{fl/fl}$ and *March5*$^{fl/fl;Lyz\text{-}Cre}$ BMDMs were infected with (D) *Citrobacter rodentium* (20 MOI, 40 MOI and 80 MOI), (E) *Salmonella typhimurium* (1 MOI, 5 MOI and 10 MOI), and (F) *Psedumonas aeruginosa* (1 MOI, 10 MOI and 20 MOI). Secretions of caspase-1 in BMDMs infected for 12 h were measured (D–F), and the cell pellet was used for western blotting to detect the activation of NLRP3 inflammasome (G) in response to bacterial infection. Values, $^{***}P < 0.001$, $^{****}P < 0.0001$ (two-tailed Student's *t*-test). Data were expressed as the mean ± SEM.

Source data are available online for this figure.

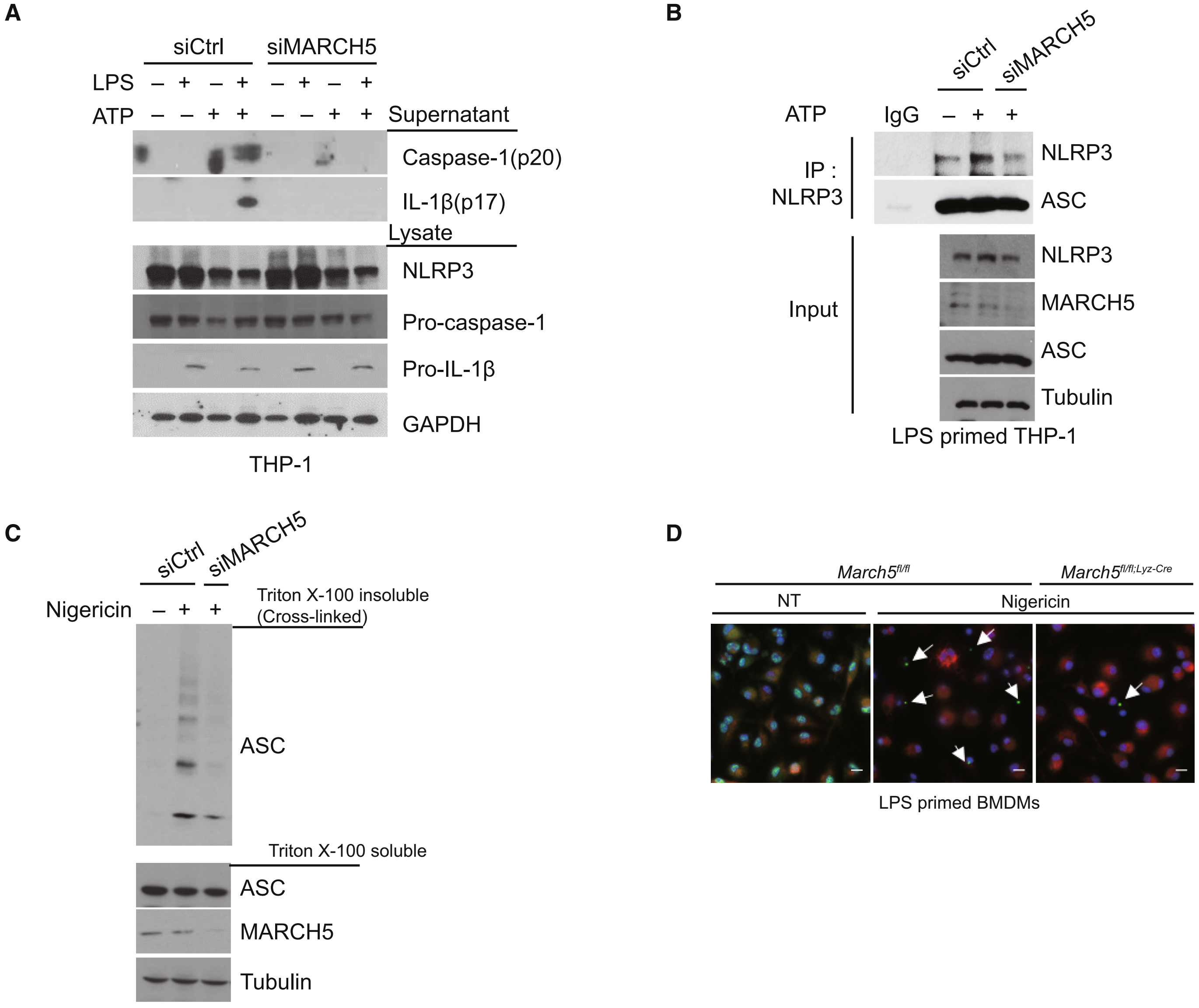

Figure EV4.

© 2023 The Authors

Figure EV4. MARCH5 is essential for activating the NLRP3 inflammasome.

A THP-1 cells transfected with siControl or siMARCH5 were untreated or treated with LPS alone for 4 h, ATP alone for 45 min, or LPS and ATP together.

B THP-1 cells transfected with siControl or siMARCH5 were treated with ATP for 30 min and then subjected to immunoprecipitation with ASC antibody. The levels of the indicated proteins were assessed by western blotting.

C THP-1 cells transfected with siControl or siMARCH5 were primed with LPS for 4 h and treated with 15 μM nigericin for 30 min. Triton X-100 insoluble pellets were cross-linked with DSS and immunoblotted with the indicated antibodies to assess ASC oligomerization.

D Fluorescence microscopy images of ASC specks in LPS-primed $March5^{fl/fl}$ and *March5*$^{fl/fl;Lyz\text{-}Cre}$ BMDMs treated with nigericin (15 μM) for 15–20 min. ASC, green; Mitochondria, red; Nuclei, blue. White arrows indicate ASC specks. Bars, 10 μm. Independent experiments were repeated at least three times.

Source data are available online for this figure.

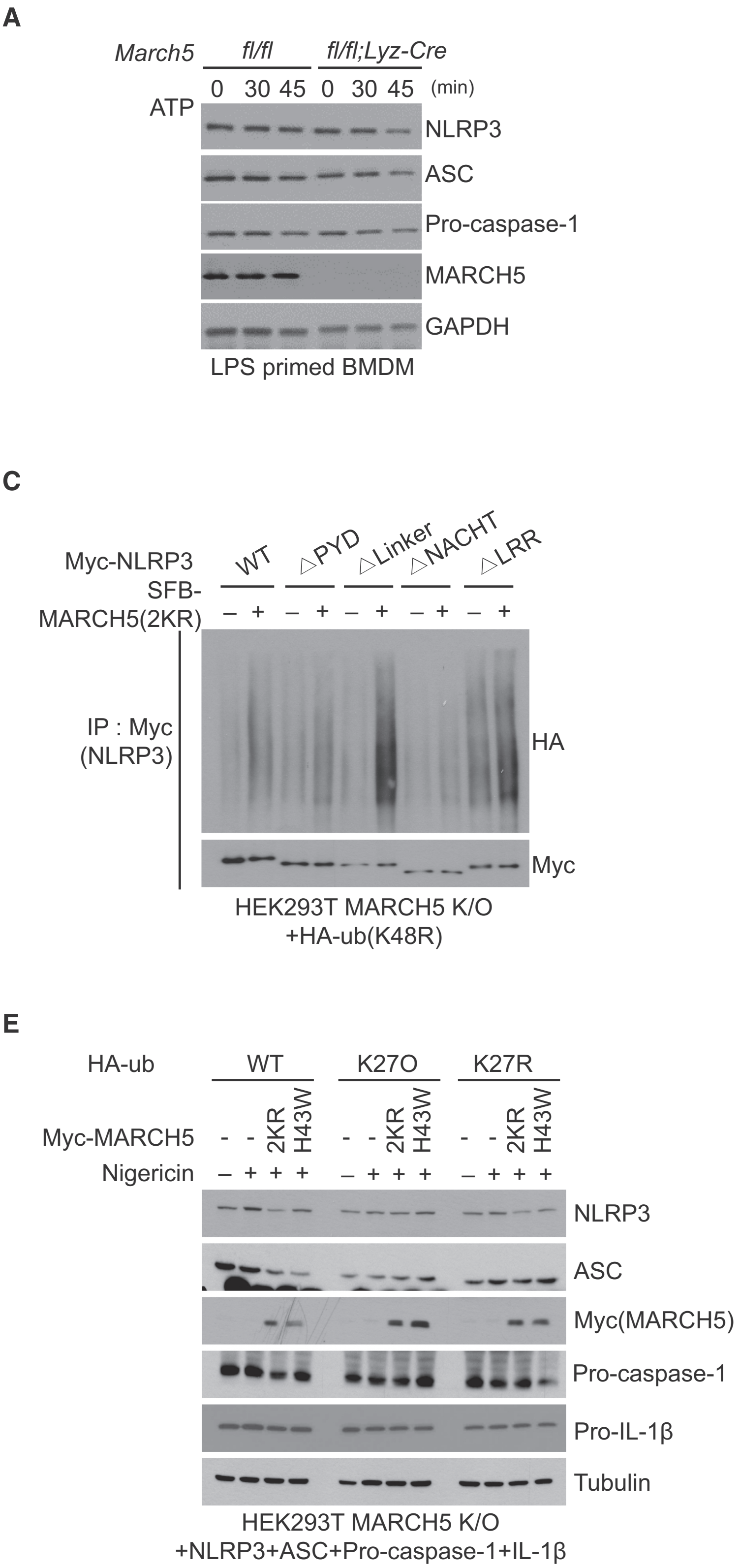

Figure EV5.

 © 2023 The Authors

◀ **Figure EV5. K27 ubiquitination and activation of NLRP3 by MARCH5.**

A *March5*$^{fl/fl}$ and March5$^{fl/fl;Lyz\text{-}Cre}$ BMDMs were primed with LPS for 4 h and were stimulated without or with 5 mM ATP at the indicated time points. Whole-cell lysates were analyzed via western blotting using the indicated antibodies.

B MARCH5 KO HEK293T cells were cotransfected with HA-ub (K48R) mutant and NLRP3 without or with Myc-MARCH5 2KR or Myc-MARCH5 H43W. After treatment with nigericin for 30 min of the LPS treatment for 4 h, the cell lysates were subjected to immunoprecipitation with anti-FLAG M2 beads. An HA antibody was used to assess NLRP3 ubiquitination.

C HEK293T MARCH5 KO cells were cotransfected with HA-ub (K48R) and NLRP3 WT or NLRP3 truncated mutants with or without SFB-MARCH5 (2KR). After treatment with LPS and nigericin, the cell lysates were subjected to immunoprecipitation with an anti-Myc antibody overnight, and NLRP3 ubiquitination was assessed via western blotting by using an anti-HA antibody.

D HEK293T MARCH5 KO cells were transfected HA-ub (K27O) with NLRP3 WT and truncated mutants. After stimulation with LPS and nigericin, cell lysates were immunoprecipitated with anti-Myc antibody. The HA antibody detected ubiquitination in the subjects.

E The NLRP3 inflammasome was reconstituted in HEK293T MARCH5 KO cells expressing ASC, pro-caspase 1, and IL-1β with HA-ub WT, K27O mutant, or K27R mutant NLRP3. Additionally, cells were cotransfected with or without Myc-MARCH5 2KR or the Myc-MARCH5 H43W mutant. After stimulation with LPS for 4 h and nigericin for 30 min, IL-1β secretion was quantitated using ELISA, and the lysates were detected by western blotting with the indicated antibodies.

Source data are available online for this figure.
