# Supplementary figures and images for: MARCH5‐dependent NLRP3 ubiquitination is required for mitochondrial NLRP3‐NEK7 complex formation and NLRP3 inflammasome activation
Source: EMBO J. 2023 Aug 14;42(19):e113481. doi: 10.15252/embj.2023113481 (PMC10548170; doi:10.15252/embj.2023113481)

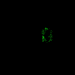

Supplement: Supplementary file 7 — Source Data for Figure 3 [file EMBJ-42-e113481-s011.zip › EMBOJ-2023-113481-Figure 3/E/March5flfl,Lyz-cre_Nigericin_ASC.png]

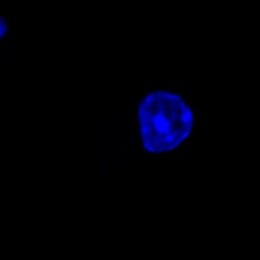

Supplement: Supplementary file 7 — Source Data for Figure 3 [file EMBJ-42-e113481-s011.zip › EMBOJ-2023-113481-Figure 3/E/March5flfl,Lyz-cre_Nigericin_DAPI.jpg]

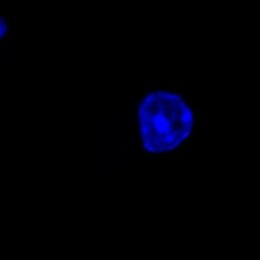

Supplement: Supplementary file 7 — Source Data for Figure 3 [file EMBJ-42-e113481-s011.zip › EMBOJ-2023-113481-Figure 3/E/March5flfl,Lyz-cre_Nigericin_DAPI.tif]

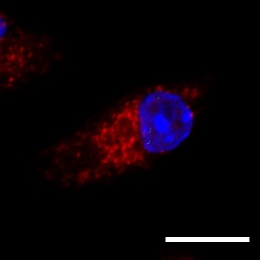

Supplement: Supplementary file 7 — Source Data for Figure 3 [file EMBJ-42-e113481-s011.zip › EMBOJ-2023-113481-Figure 3/E/March5flfl,Lyz-cre_Nigericin_MERGE.png]

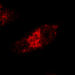

Supplement: Supplementary file 7 — Source Data for Figure 3 [file EMBJ-42-e113481-s011.zip › EMBOJ-2023-113481-Figure 3/E/March5flfl,Lyz-cre_Nigericin_Mitored.png]

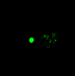

Supplement: Supplementary file 7 — Source Data for Figure 3 [file EMBJ-42-e113481-s011.zip › EMBOJ-2023-113481-Figure 3/E/March5flfl_Nigericin_ASC.png]

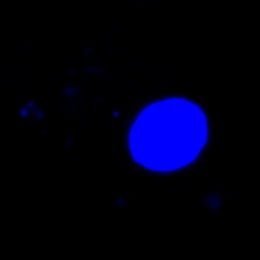

Supplement: Supplementary file 7 — Source Data for Figure 3 [file EMBJ-42-e113481-s011.zip › EMBOJ-2023-113481-Figure 3/E/March5flfl_Nigericin_DAPI.png]

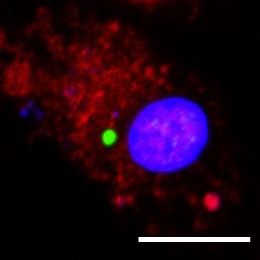

Supplement: Supplementary file 7 — Source Data for Figure 3 [file EMBJ-42-e113481-s011.zip › EMBOJ-2023-113481-Figure 3/E/March5flfl_Nigericin_Merge.png]

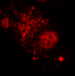

Supplement: Supplementary file 7 — Source Data for Figure 3 [file EMBJ-42-e113481-s011.zip › EMBOJ-2023-113481-Figure 3/E/March5flfl_Nigericin_Mitored.png]

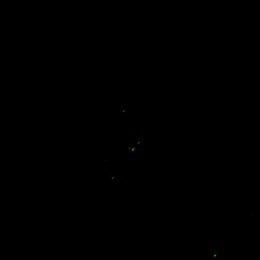

Supplement: Supplementary file 7 — Source Data for Figure 3 [file EMBJ-42-e113481-s011.zip › EMBOJ-2023-113481-Figure 3/E/NT_ASC.jpg]

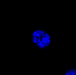

Supplement: Supplementary file 7 — Source Data for Figure 3 [file EMBJ-42-e113481-s011.zip › EMBOJ-2023-113481-Figure 3/E/NT_DAPI.png]

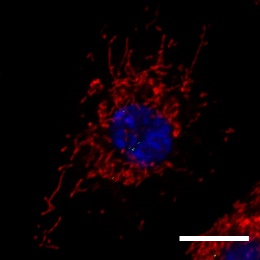

Supplement: Supplementary file 7 — Source Data for Figure 3 [file EMBJ-42-e113481-s011.zip › EMBOJ-2023-113481-Figure 3/E/NT_Merge.png]

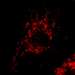

Supplement: Supplementary file 7 — Source Data for Figure 3 [file EMBJ-42-e113481-s011.zip › EMBOJ-2023-113481-Figure 3/E/NT_Mito-red.png]

Figure 3

A

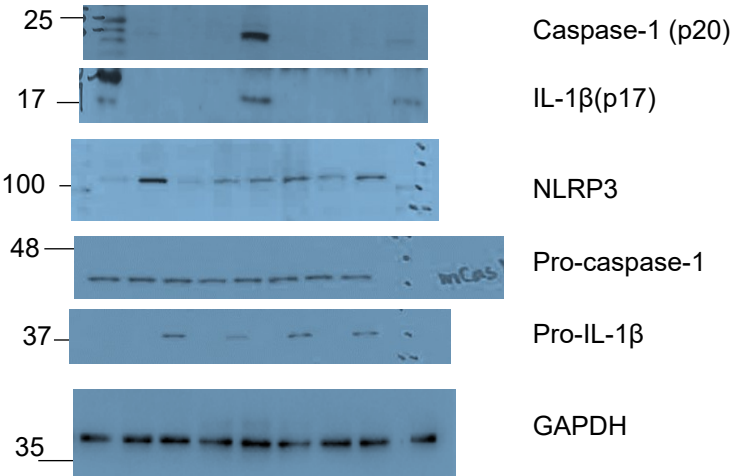

C

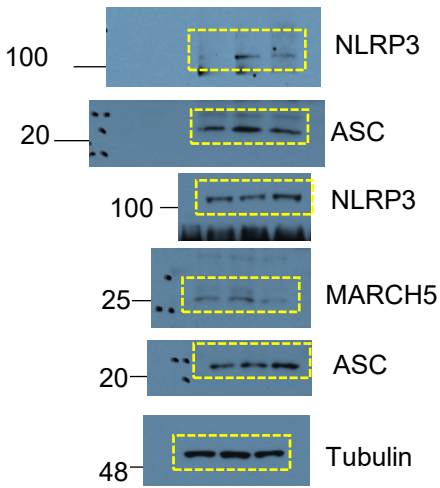

D

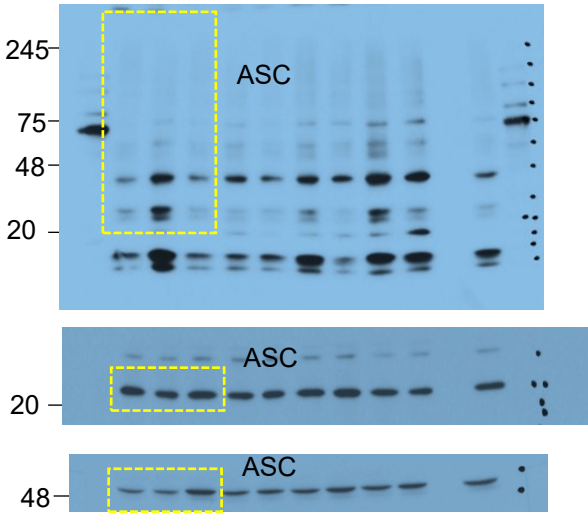

Supplement: Supplementary file 7 — Source Data for Figure 3 [file EMBJ-42-e113481-s011.zip › EMBOJ-2023-113481-Figure 3/Western blot(Figure 3).pdf]

**Figure 4**

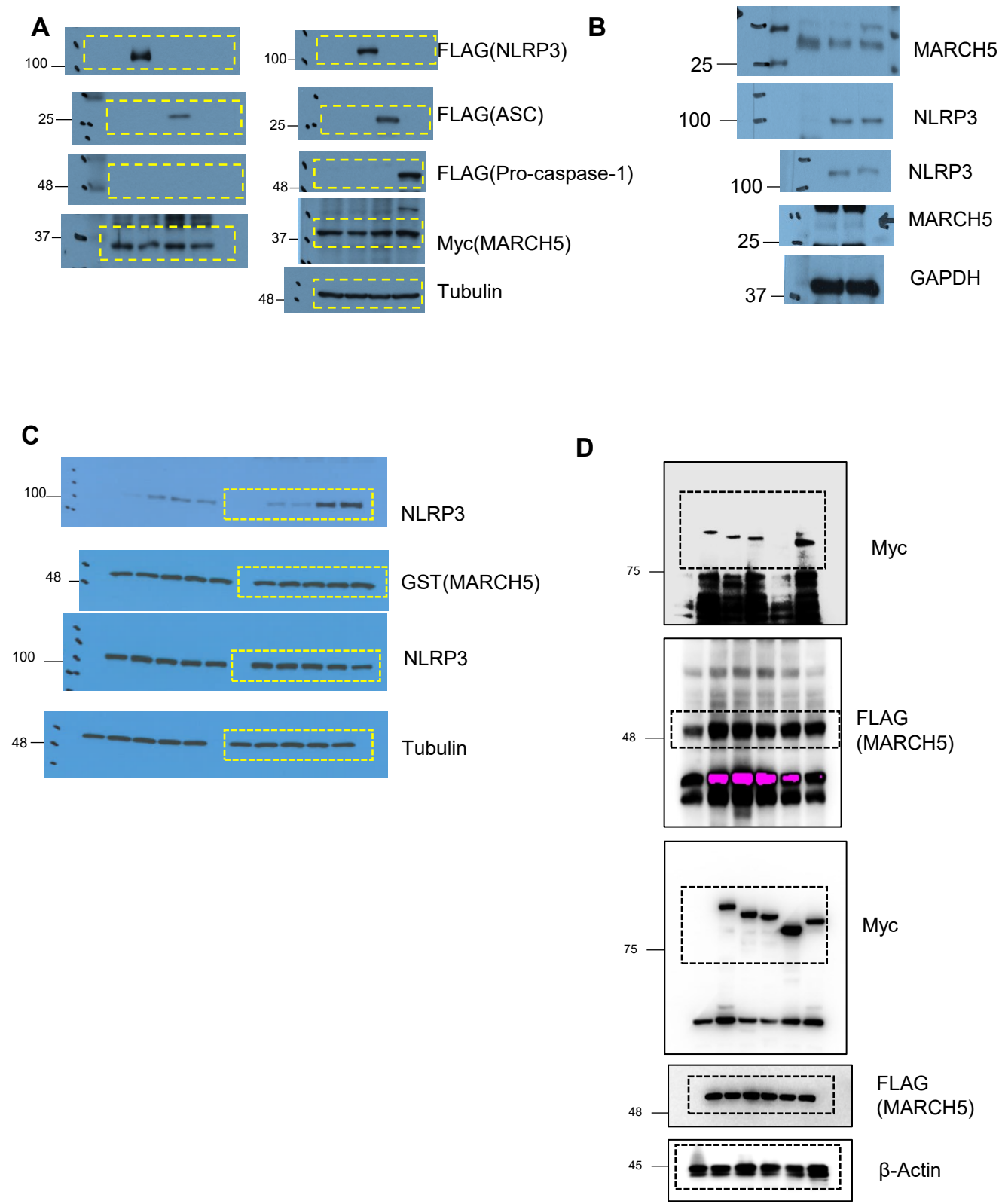

Supplement: Supplementary file 8 — Source Data for Figure 4 [file EMBJ-42-e113481-s005.zip › EMBOJ-2023-113481-Figure 4/Western blot.pdf]

**Figure 5**

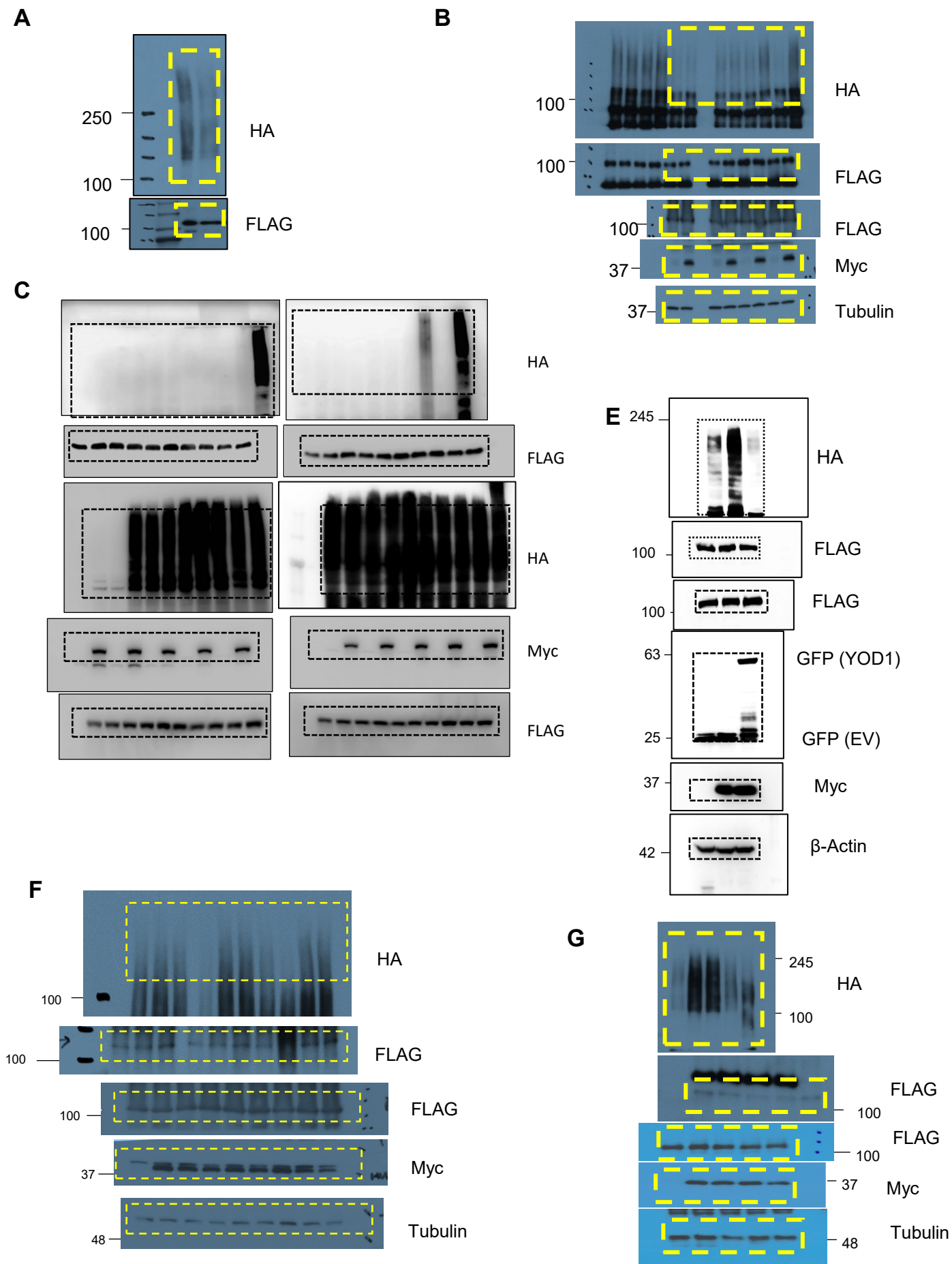

Supplement: Supplementary file 9 — Source Data for Figure 5 [file EMBJ-42-e113481-s010.zip › EMBOJ-2023-113481-Figure 5/Figure 5.pdf]

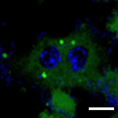

Supplement: Supplementary file 10 — Source Data for Figure 6 [file EMBJ-42-e113481-s009.zip › EMBOJ-2023-113481R-Figure 6 Source Data/A/siCtrl_Merge.png]

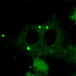

Supplement: Supplementary file 10 — Source Data for Figure 6 [file EMBJ-42-e113481-s009.zip › EMBOJ-2023-113481R-Figure 6 Source Data/A/siCtrl_NLRP3.png]

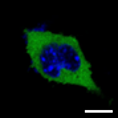

Supplement: Supplementary file 10 — Source Data for Figure 6 [file EMBJ-42-e113481-s009.zip › EMBOJ-2023-113481R-Figure 6 Source Data/A/siMarch5_Merge.png]

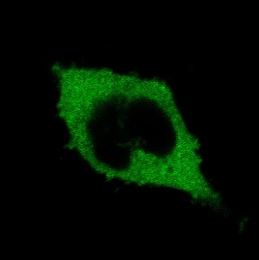

Supplement: Supplementary file 10 — Source Data for Figure 6 [file EMBJ-42-e113481-s009.zip › EMBOJ-2023-113481R-Figure 6 Source Data/A/siMarch5_NLRP3.png]

**Figure 6**

**A**

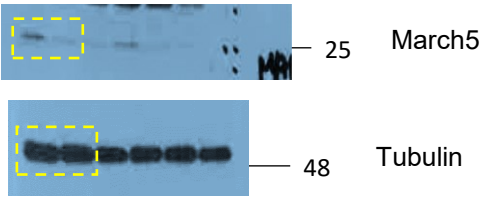

**B**

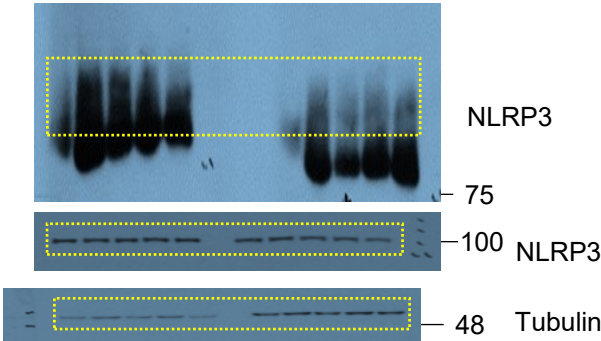

**C**

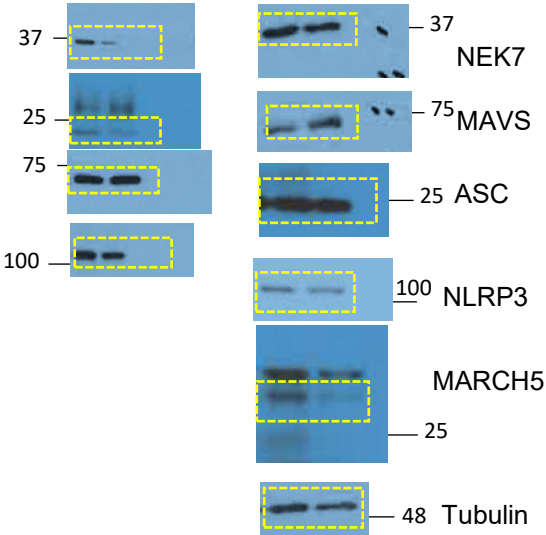

**D**

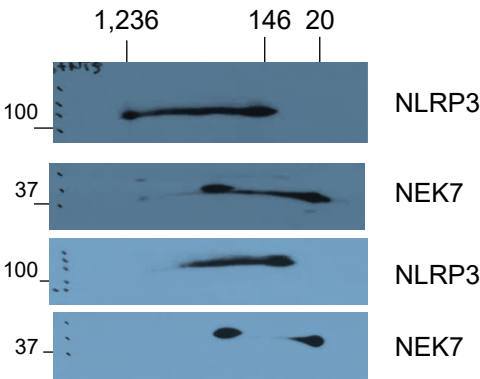

**E**

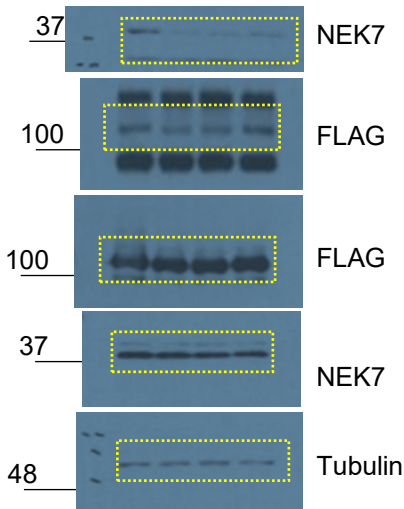

**F**

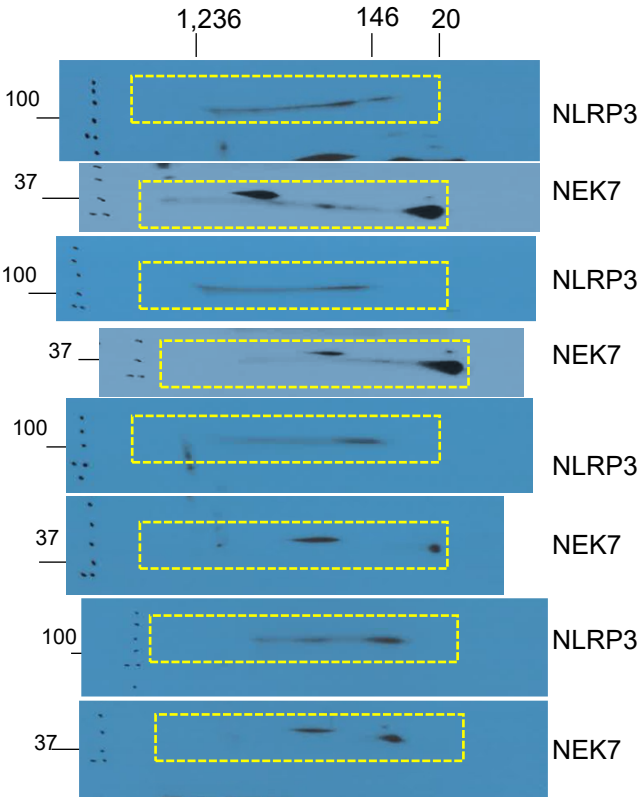

Supplement: Supplementary file 10 — Source Data for Figure 6 [file EMBJ-42-e113481-s009.zip › EMBOJ-2023-113481R-Figure 6 Source Data/Figure 6.pdf]

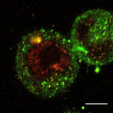

Supplement: Supplementary file 10 — Source Data for Figure 6 [file EMBJ-42-e113481-s009.zip › EMBOJ-2023-113481R-Figure 6 Source Data/G/siCtrl_Merge.png]

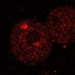

Supplement: Supplementary file 10 — Source Data for Figure 6 [file EMBJ-42-e113481-s009.zip › EMBOJ-2023-113481R-Figure 6 Source Data/G/siCtrl_NEK7.png]

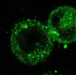

Supplement: Supplementary file 10 — Source Data for Figure 6 [file EMBJ-42-e113481-s009.zip › EMBOJ-2023-113481R-Figure 6 Source Data/G/siCtrl_NLRP3.png]

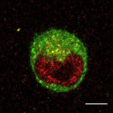

Supplement: Supplementary file 10 — Source Data for Figure 6 [file EMBJ-42-e113481-s009.zip › EMBOJ-2023-113481R-Figure 6 Source Data/G/siMarch5_Merge.png]

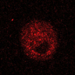

Supplement: Supplementary file 10 — Source Data for Figure 6 [file EMBJ-42-e113481-s009.zip › EMBOJ-2023-113481R-Figure 6 Source Data/G/siMarch5_NEK7.png]

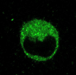

Supplement: Supplementary file 10 — Source Data for Figure 6 [file EMBJ-42-e113481-s009.zip › EMBOJ-2023-113481R-Figure 6 Source Data/G/siMarch5_NLRP3.png]

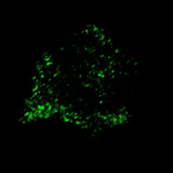

Supplement: Supplementary file 11 — Source Data for Figure 7 [file EMBJ-42-e113481-s004.zip › EMBOJ-2023-113481-Figure 7/A/K324R_ASC.png]

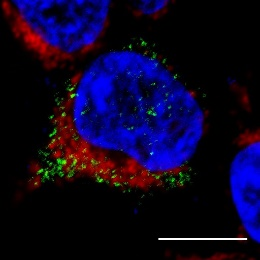

Supplement: Supplementary file 11 — Source Data for Figure 7 [file EMBJ-42-e113481-s004.zip › EMBOJ-2023-113481-Figure 7/A/K324R_Merge.png]

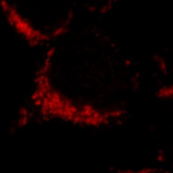

Supplement: Supplementary file 11 — Source Data for Figure 7 [file EMBJ-42-e113481-s004.zip › EMBOJ-2023-113481-Figure 7/A/K324R_Mitored.png]

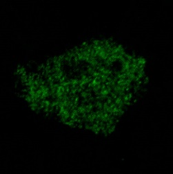

Supplement: Supplementary file 11 — Source Data for Figure 7 [file EMBJ-42-e113481-s004.zip › EMBOJ-2023-113481-Figure 7/A/K430R_ASC.png]

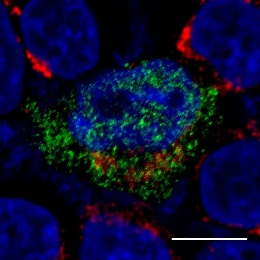

Supplement: Supplementary file 11 — Source Data for Figure 7 [file EMBJ-42-e113481-s004.zip › EMBOJ-2023-113481-Figure 7/A/K430R_Merge.png]

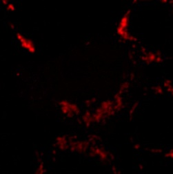

Supplement: Supplementary file 11 — Source Data for Figure 7 [file EMBJ-42-e113481-s004.zip › EMBOJ-2023-113481-Figure 7/A/K430R_Mitored.png]

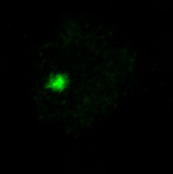

Supplement: Supplementary file 11 — Source Data for Figure 7 [file EMBJ-42-e113481-s004.zip › EMBOJ-2023-113481-Figure 7/A/WT_ASC.png]

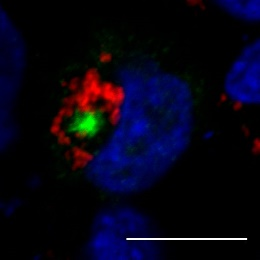

Supplement: Supplementary file 11 — Source Data for Figure 7 [file EMBJ-42-e113481-s004.zip › EMBOJ-2023-113481-Figure 7/A/WT_Merge.png]

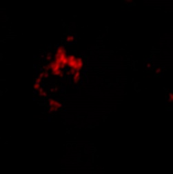

Supplement: Supplementary file 11 — Source Data for Figure 7 [file EMBJ-42-e113481-s004.zip › EMBOJ-2023-113481-Figure 7/A/WT_Mitored.png]

Figure 7

B

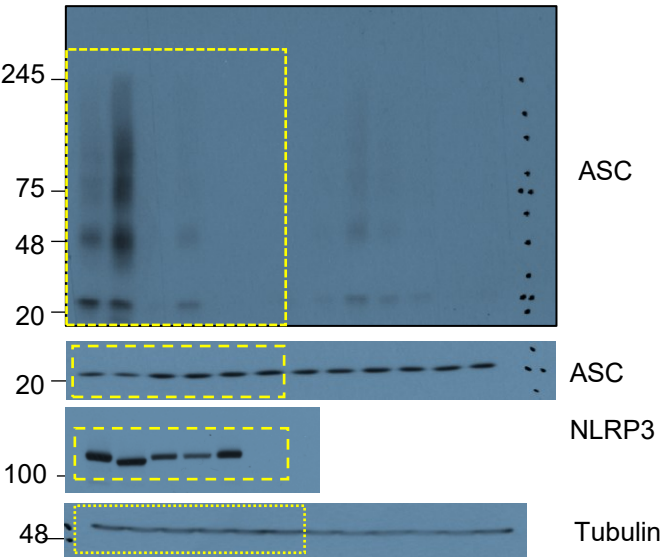

C

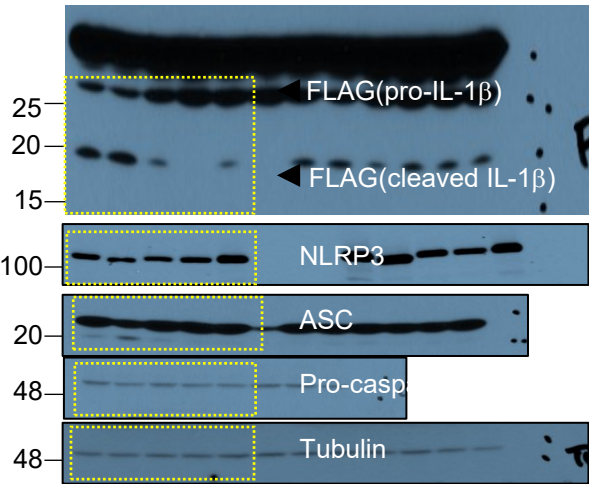

Supplement: Supplementary file 11 — Source Data for Figure 7 [file EMBJ-42-e113481-s004.zip › EMBOJ-2023-113481-Figure 7/Figure 7(western blot).pdf]
