# Supplementary material for: MARCH5‐dependent NLRP3 ubiquitination is required for mitochondrial NLRP3‐NEK7 complex formation and NLRP3 inflammasome activation
Source: EMBO J. 2023 Aug 14;42(19):e113481. doi: 10.15252/embj.2023113481 (PMC10548170; doi:10.15252/embj.2023113481)
Supplement: Supplementary file 12 [file EMBJ-42-e113481-s003.pdf]

THE EMBO JOURNAL

MARCH5-dependent NLRP3 ubiquitination is required for mitochondrial NLRP3-NEK7 complex formation and NLRP3 inflammasome activation

Yeon-Ji Park, Niranjan Dodantenna, Yonghyeon Kim, Tae-Hwan Kim, Ho-Soo Lee, Young-Suk Yoo, June Heo, Jae-Ho Lee, Myung-Hee Kwon, Ho Chul Kang, Jong-Soo Lee, and Hyeseong Cho
DOI: 10.15252/embj.2023113481

Corresponding author(s): Hyeseong Cho (hscho@ajou.ac.kr) , Jong-Soo Lee (jongsool@cnu.ac.kr)

Review Timeline:

Submission Date:	11th Jan 23
Editorial Decision:	22nd Feb 23
Revision Received:	16th May 23
Editorial Decision:	18th Jul 23
Revision Received:	20th Jul 23
Accepted:	23rd Jul 23

Editor: Karin Dumstrei

Transaction Report:

(Note: With the exception of the correction of typographical or spelling errors that could be a source of ambiguity, letters and reports are not edited. Depending on transfer agreements, referee reports obtained elsewhere may or may not be included in this compilation. Referee reports are anonymous unless the Referee chooses to sign their reports.)

1st Editorial Decision 22nd Feb 2023

Dear Prof. Cho,

Thank you for submitting your manuscript to The EMBO Journal. Your study has now been seen by three referees and their comments are provided below.

As you can see from the comments, the referees find the analysis interesting and suitable for publication here. They raise several different concerns that should be resolved. I would therefore like to invite you to submit a revised manuscript.

It would be helpful to discuss the raised points further and I am available to do so via email or video. Let me know when a good time for you is.

When preparing your letter of response to the referees' comments, please bear in mind that this will form part of the Review Process File, and will therefore be available online to the community. For more details on our Transparent Editorial Process, please visit our website: https://www.embopress.org/page/journal/14602075/authorguide#transparentprocess

I thank you for the opportunity to consider your work for publication. I look forward to discussing the revisions further with you

Yours sincerely,

Karin

Karin Dumstrei, PhD
Senior Editor
The EMBO Journal

Instructions for preparing your revised manuscript:

Please make sure you upload a letter of response to the referees' comments together with the revised manuscript.

Please also check that the title and abstract of the manuscript are brief, yet explicit, even to non-specialists.

When assembling figures, please refer to our figure preparation guideline in order to ensure proper formatting and readability in print as well as on screen:
https://bit.ly/EMBOPressFigurePreparationGuideline
See also guidelines for figure legends: https://www.embopress.org/page/journal/14602075/authorguide#figureformat

At EMBO Press we ask authors to provide source data for the main manuscript figures. Our source data coordinator will contact you to discuss which figure panels we would need source data for and will also provide you with helpful tips on how to upload and organize the files.

IMPORTANT: When you send the revision we will require
- a point-by-point response to the referees' comments, with a detailed description of the changes made (as a word file).
- a word file of the manuscript text.
- individual production quality figure files (one file per figure)
- a complete author checklist, which you can download from our author guidelines (https://www.embopress.org/page/journal/14602075/authorguide).
- Expanded View files (replacing Supplementary Information)
Please see out instructions to authors
https://www.embopress.org/page/journal/14602075/authorguide#expandedview

Please remember: Digital image enhancement is acceptable practice, as long as it accurately represents the original data and conforms to community standards. If a figure has been subjected to significant electronic manipulation, this must be noted in the figure legend or in the 'Materials and Methods' section. The editors reserve the right to request original versions of figures and the original images that were used to assemble the figure.

Further information is available in our Guide For Authors: https://www.embopress.org/page/journal/14602075/authorguide

We realize that it is difficult to revise to a specific deadline. In the interest of protecting the conceptual advance provided by the work, we recommend a revision within 3 months (23rd May 2023). Please discuss the revision progress ahead of this time with the editor if you require more time to complete the revisions.

We generally allow three months as standard revision time. As a matter of policy, competing manuscripts published during this period will not negatively impact on our assessment of the conceptual advance presented by your study. However, we request that you contact the editor as soon as possible upon publication of any related work, to discuss how to proceed.

Referee #1:

The NLRP3 inflammasome is a critical component of the innate immune system that mediates caspase-1 activation and the secretion of proinflammatory cytokines IL-1β/IL-18 in response to microbial infection and cellular damage. NLRP3 translocates to mitochondria but whether mitochondria involvement in the inflammasome assembly is unclear. In this study, the authors found that MITOL (MARCH5) interacts with NLRP3-MAVS complex and adds K27-linked polyubiquitin to NLRP3. This event provides a docking site for NEK7 binding to NLRP3, initiating NEK7-NLRP3 multimeric complex formation. They conclude that MARCH5 plays a regulatory role in NLRP3 oligomerization on mitochondria. The proposed mechanism for NLRP3 oligomerization by MARCH5-mediated ubiquitination is convincing and important for the full understanding of the mechanism for NLRP3 inflammasome assembly. I therefore believe that this paper contains sufficient interest and originality to merit publication. Several points I noticed are listed below.

Points

1. In relation to Fig. 2 and Fig. EV3, active Caspase-1 is not produced at all in MARCH5-knockout BMDMs infected with bacteria. Nevertheless, why is IL-18 increased? The authors should explain this phenomenon.

2. In Fig. 4D, the authors conclude that NACHT domain is the binding region because NLRP3-ΔNACHT failed to bind to MARCH5. Is there evidence that NLRP3-ΔNACHT can normally bind to MAVS? If NLRP3-ΔNACHT cannot bind to MAVS, it could simply be because it was not recruited to the mitochondria.

3. In Fig. 5C, KR mutants of Ubiquitin often exhibit unusual behavior. In addition, the K27-dependent ubiquitin chain is less familiar than the K63-dependent ubiquitin chain.
To identify Ubiquitin chain types, I recommend additional experiments using deubiquitinating enzymes (UbiCREST Deubiquitinase Enzyme Set, Catalog #: K-400, R&D Systems). This experiment is more convincing than the mutant experiment.

4. In relation to Fig. 5E and Fig. 7B,C, why does NLRP3-K93R show a phenotype similar to WT despite reduced ubiquitination? The authors should explain this phenomenon.

5. The authors claim that ubiquitination of NLRP3 precedes binding of NEK7 to NLRP3.
To prove that the ubiquitination of NLRP3 by MARCH5 is independent of NEK7, it is better to show that the ubiquitination of NLRP3 is not affected by NEK7 knockdown.

6. The authors argue that phosphorylation of NLRP3 and its translocation to mitochondria occurs prior to ubiquitination of NLRP3 by MARCH5. They should show that ubiquitination of NLRP3-S198A is actually reduced.

7. In their previous paper (DOI: 10.1038/ncomms8910), they showed that that Poly:IC stimulation transfers K48-linked polyubiquitin chain on MAVS by MARCH5. Does the stimulation used in this paper promote ubiquitination of MAVS by MARCH5? If not, how do MARCH5 selectively ubiquitinates NLRP3, not MAVs? If ubiquitination does occur, is it possible that the authors have misinterpreted the ubiquitination signal of MAVS bound to NLRP3 as ubiquitination of NLRP3? They should provide evidence to deny this possibility.

(Typos)
1. "This analysis predicted seven ubiquitination sites on Lys (K9, K192, K194, K324, K430, K689, K696) of NLRP3." K93 is correct, not K9.
2. "In this experiment, we found that MARCH5 2KR but not MARCH5 H43W enhanced NLRP3 polyubiquitination independent of K48-linked ubiquitin (Fig S5B)." Fig EV5B is correct, not Fig S5B.

Referee #2:

In the manuscript "MARCH5-dependent NLRP3 ubiquitination is an essential step for NEK7 docking on the mitochondria", Park et al. investigate the role of MARCH5, a mitochondrial E3 ligase, in innate immunity. They build this story on in vivo observations on conditional MARCH5 knockout mice with deletion in cells of the myeloid lineage and observe protection in infection and LPS-induced shock models. They then try to connect this to a role of MARCH5 in NLRP3 inflammasome activation. They propose MARCH5 ubiquitinates NLRP3 on the mitochondrial membrane. Upon MAVS binding, this K27-polyubiquitination should trigger NEK7 binding to NLRP3, causing assembly of the NLRP3 inflammasome with the recruitment of adaptor protein ASC and pro-Caspase1. The important role of mitochondria and the ubiquitin system in NLRP3 inflammasome is well establish, yet this is the first report on a role MARCH5 therein. Although the story is potentially interesting, there are some conceptual and experimental concerns.

Major points:

1. The role of MAVS in NLRP3 activation is highly controversial. We would recommend to revisit this and either show a specific involvement here or potentially remove this aspect if not critical to the story.
2. NEK7 is not absolutely required for NLRP3 activation. Do the authors think that MARCH5 is also involved in the NEK7-independent mechanism of NLRP3 activation?
3. At some points, e.g., Fig 2., a direct comparison to NLRP3 knockout cells should be included to estimate the degree of MARCH5-dependency.
4. Salmonella is not a typical NLRP3 activator and also triggers other inflammasomes. Do the authors think that MARCH5 could also impact other inflammasomes and how would this relate to mitochondria?
5. Throughout the paper, inflammasome activation seems to be suboptimal with low IL-1β or LDH values and a low cell frequency of inflammasome activation. This not only raises doubts about the experimental prowess, but also the concern that the phenotype is only observed under suboptimal stimulation conditions but lost under optimal conditions. Dose/response and time-course experiments that reach the plateau should be shown, e.g., with nigericin. (LPS and nigericin concentrations used throughout the article are relatively high, maybe too high). Frequency as in Fig. 7 should also be included in earlier data sets such as in Fig. 2. FACS data for pyroptosis could also be useful is this respect.
6. Why are different MOI used for different read-outs for the three bacterial strains, as in Fig 2C? The IL-18 levels detected here are quite high, while the concentrations of the other cytokines measured here are very low.
7. Why is the MARCH5 phenotype seen for nigericin and ATP much less clear than for live pathogens? We would expect the opposite.
8. UbPred predicted only 7 ubiquitination sites on NLRP3 while there are more sites reported in the literature already. Why did the others not show up in the prediction and why were not considered in the experimental analysis? Could a proteomics approach have been used to determine Ub sites?
9. At the end of the discussion, the authors propose to regulate MARCH5 activity as a new therapeutic strategy. Potential side effects should be discussed. What is the phenotype of MARCH5 full knockouts? What phenotype could be expected when deleting MARCH5 in adults, e.g., using ROSA26-ERT2-Cre mice? What effect would global inhibition of MARCH5 have in mice and humans? How likely is it to be able to develop a strategy that only affects the effect of MARCH5 on NLRP3 but not its other functions?
10. Westerns blots for Casp1 activation and to exclude effects on pro-IL-1β levels are missing throughout. Data in Fig 3A suggests an effect of MARCH5-deficiency on NLRP3 expression. This should be verified or excluded.
11. The THP-1 data in Fig EV4A is not convincing, cleaved Casp1 bands are blurry, and the IL-1β band in the knockout seems unaltered. THP-1 cells should express NLRP3 and pro-IL-1β without LPS due to differentiation with PMA. There is a typo also in this panel. Nigericin might work better than ATP in THP-1 cells.

Specific points:

12. Fig 3B: in the casp1 activity assay, RLU levels are really low. Was it tested with other activators? Is there data for THP-1 cells?
13. Fig 3: C,D: which time points were used for ATP and Nigericin treatment? 45 min as for ATP treatment in Fig 3A?
14. Fig 3D, why is there an ASC dimer background in the untreated condition?
15. Fig 3E, why such a low frequency with nigericin or ATP? Were cells transfected with Poly(dA:dT) not primed with LPS?
16. Fig 4D, we see a band there for deltaNACHT.
17. Fig 5D, the labelling of the X-axis is difficult to understand. The figure legend should outline in more detail what the labelling is referring it. There are no error bars for the condition MARCH KO HEK293T expressing K27O and transfected with Myc-MARCH5 H43W mutant? Prism settings? Nomenclature with K27O and K27R is misleading. K27R is never explained (only K48R previously).
18. Fig 5E, what about K93R, why was this not followed up? How can mutation of two individual sites each lead to an almost complete loss in ubiquitination? There is a typo on predicted Ub Sites: K189 is written instead of K689 on the graph.
19. In the first part of the results where MARCH5 immune-modulatory functions are investigated, the authors claim no significant difference between TNF and IL-6 levels in P.aeruginosa infected mice and control group. In the endotoxemia-induced septic shock model, they then say that the results were "inconsistent" with the previous model. However, they come to the same conclusion of no difference between TNF and IL-6 levels. Please check or specify what is meant with "inconsistent" here.
20. Typo in "MARCH5 promotes NLRP3 activation via K27-linked polyubiquitination of the Lys324 and Lys430 residues of NLRP3" paragraph: figure EV5B is referred to as S5B.
21. Fig EV5E: why is there no pro-IL-1β in the blot shown in cell lysate or IL-1β p17 in supernatant to confirm ELISA results?
22. In the discussion: typo at the sentence "Thus, the data suggest that NLRR3 recruitment to mitochondria by MAVS precedes its interaction with MARCH5" NLRR3 instead of NLRP3.
23. In the discussion: "We observed that cells expressing the NLRP3-S194A mutant also failed to form the multimeric NEK7-

NLRP3 complex (Figs 6E and F)." However, experiments were made in human cells (S198A mutant) and not in mouse.
24. Material section missing reference for anti-HA antibody.
25. The figure legends are inconsistent at times (e.g., time points missing)

Referee #3:

Mitochondria are known to be intensely involved in inflammasome activation as they increase the production of reactive oxygen species (ROS) and release oxidized fragments of mitochondrial DNA into the cytosol, which stimulates inflammasome activation. The manuscript by Park et al. now describes that the human inflammasomal protein NLRP3 is ubiquitinated by the mitochondria-associated E3-ligase MARCH5. Using truncation and mutagenesis studies the authors show that MARCH5 interacts with the NACHT domain of NLRP3 and promotes K27-linked polyubiquitination of K324 and K430 residues of NLRP3. MARCH5 knockout mice failed to secrete IL-1b and IL-18 from macrophages upon bacterial infection and exhibited an attenuated mortality rate. Ubiquitination at the two sites in the NACHT domain is required for NEK7 binding and NLRP3 oligomerization, suggesting that the E3 ligase MARCH5 is an important regulator of NLRP3 function.

Overall, the authors present a comprehensive study, starting with an analysis of the E3 ligase in bacterial activation assays (Figs 1, 2), the assignment to the activation mechanism of NLRP3 (Fig. 3), a mapping to the NACHT domain of NLRP3 (Fig. 4), the molecular characterization of K27-ubiquitination and identification of two (three) sites on the target protein (Fig. 5), the requirement of NLRP3 ubiquitination for NEK7 binding and activation on a molecular level (Fig. 6) and on a functional level, analysing IL-1beta release (Fig. 7).

The experimental part seems thoroughly performed, but the text is rather heterogeneously written. While the Abstract and the Introduction sections need serious editing, the Results and Discussion sections are well written. I have a couple of comments to the present version of the manuscript.

Comments:
In the cellular ubiquitination assay using K-to-R mutants the signal loss for the K93R mutant appears as strong as for the K430R mutant, and indeed much more than for the K324R mutant. It is well taken that the E3 ligase many interact with the NACHT domain, but why did the authors omit the K93 site in the following? Interestingly, the K93R mutant shows even stronger ASC dimer and oligomerization bands in Figure 7B. Could this be a negative regulation, such that K93-Ub prevents inflammasome formation, while K430-Ub stimulates it?

How do the identified ubiquitination sites fit to the recent structural data of the active NLRP3-NEK7 inflammasome (Xiao et al., 2023)? Is there a molecular rational for the diminished NEK7 binding of the K324 and K430 arginine mutants (Fig. 6E)? And how do the sites align to the resting state of NLRP3 (Andreeva et al, Hochheiser et al)? Could ubiquitination lead to an opening of the NACHT domain, as described in the Discussion section as "rotational activation" (page 13, last paragraph).

Minor:
Page number (or line numbers) are missing, which is bad.

Introduction, page 4, second line: "In the resting state, inactive NLRP3 retails its folded structure, but it convers to the unfolded form ..." Where has this been shown? Please give a citation for this statement. It now rather appears, that inactive NLRP3 is an oligomer (decamer, PDB ID: 7pzc) as is the active NLRP3-NEK7 complex (hetero-decamer, PDB ID: 8ej4).

Figure 4A and 4B: the lanes are not well labelled, see, e.g. the "-" and "+" in panel B. Same holds for Fig. 5E.

Discussion: "NLRP3 phosphorylation at Ser194 is a key priming event ..." Please use human NLRP3 numbering throughout. This site was designated as S198 elsewhere in the manuscript (Fig. 7, ...).

Karin Dumstrei, PhD
Senior Editor
The EMBO Journal

May 16, 2023

Manuscript ID: EMBOJ-2023-113481

Manuscript Title: MARCH5-dependent NLRP3 ubiquitination is an essential step for NEK7 docking on the mitochondria

Dear Dr. Dumstrei,

We wish to thank you and three referees for taking the time to analyze our manuscript. We are also grateful that “*EMBO Journal*” is interested in publishing our work after proper revision.

Please find a point-by-point response to the referees.

Responses to the Reviewers' comments (comments from reviewers in **black,** responses to the reviewers in **blue)**

Referee #1:

The NLRP3 inflammasome is a critical component of the innate immune system that mediates caspase-1 activation and the secretion of proinflammatory cytokines IL-1β/IL-18 in response to microbial infection and cellular damage. NLRP3 translocates to mitochondria but whether mitochondria involvement in the inflammasome assembly is unclear. In this study, the authors found that MITOL (MARCH5) interacts with NLRP3-MAVS complex and adds K27-linked polyubiquitin to NLRP3. This event provides a docking site for NEK7 binding to NLRP3, initiating NEK7-NLRP3 multimeric complex formation. They conclude that MARCH5 plays a regulatory role in NLRP3 oligomerization on mitochondria. The proposed mechanism for NLRP3 oligomerization by MARCH5-mediated ubiquitination is convincing and important for the full understanding of the mechanism for NLRP3 inflammasome assembly. I therefore believe that this paper contains sufficient interest and originality to merit publication. Several points I noticed are listed below.
→ We would like to thank the reviewer for evaluating our work as well as for allowing us to improve the study. We have thoughtfully revised the manuscript and addressed the reviewer's comments below.

Points
1. In relation to Fig. 2 and Fig. EV3, active Caspase-1 is not produced at all in MARCH5-knockout BMDMs infected with bacteria. Nevertheless, why is IL-18 increased? The authors should explain this phenomenon.

→As pointed by the referee, IL-18 levels appeared to be increased at the condition of high MOI of bacterial infection (Fig. 2 and Fig. EV3). To verify them we carried out the same experiments and obtained similar results as shown below.

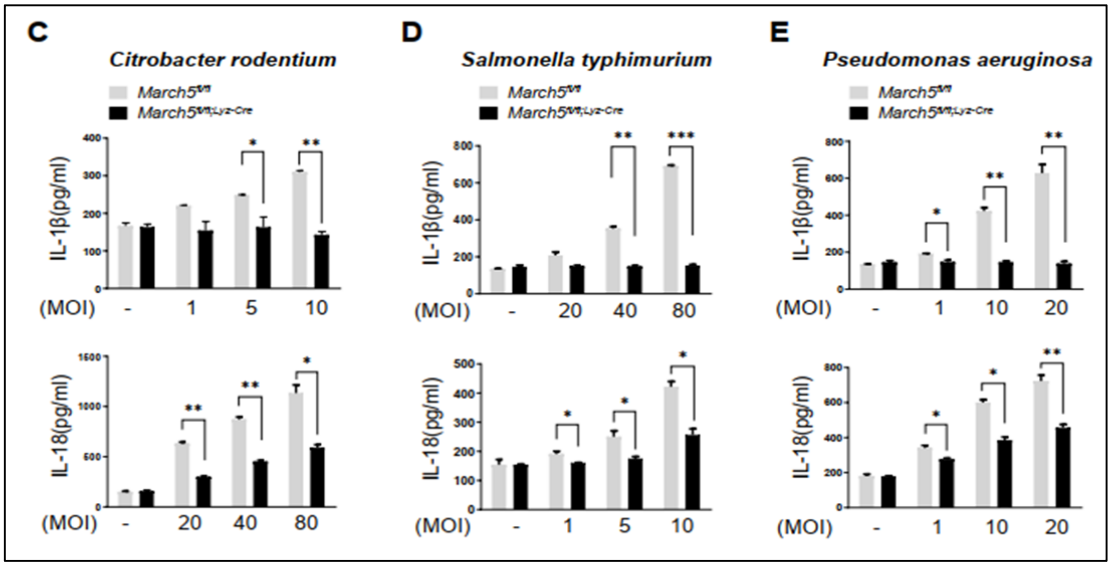

Figure 2C-E (original data)

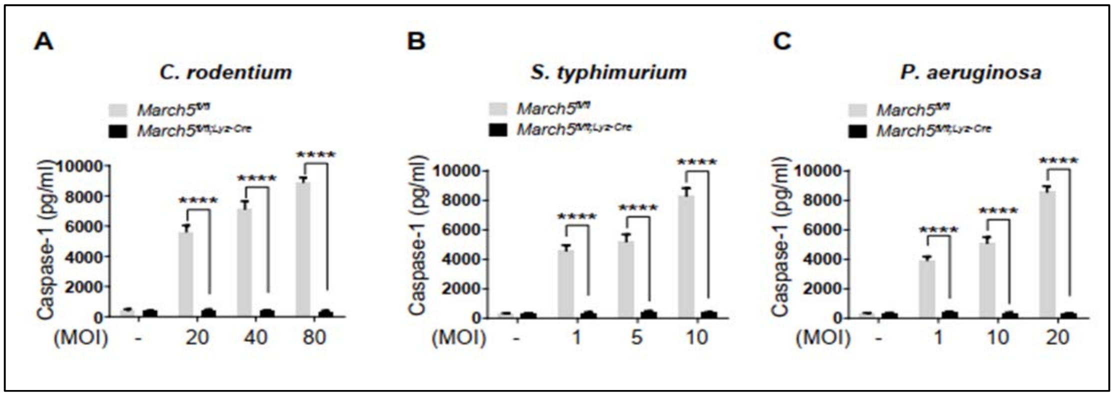

Figure EV3A-C (original data)

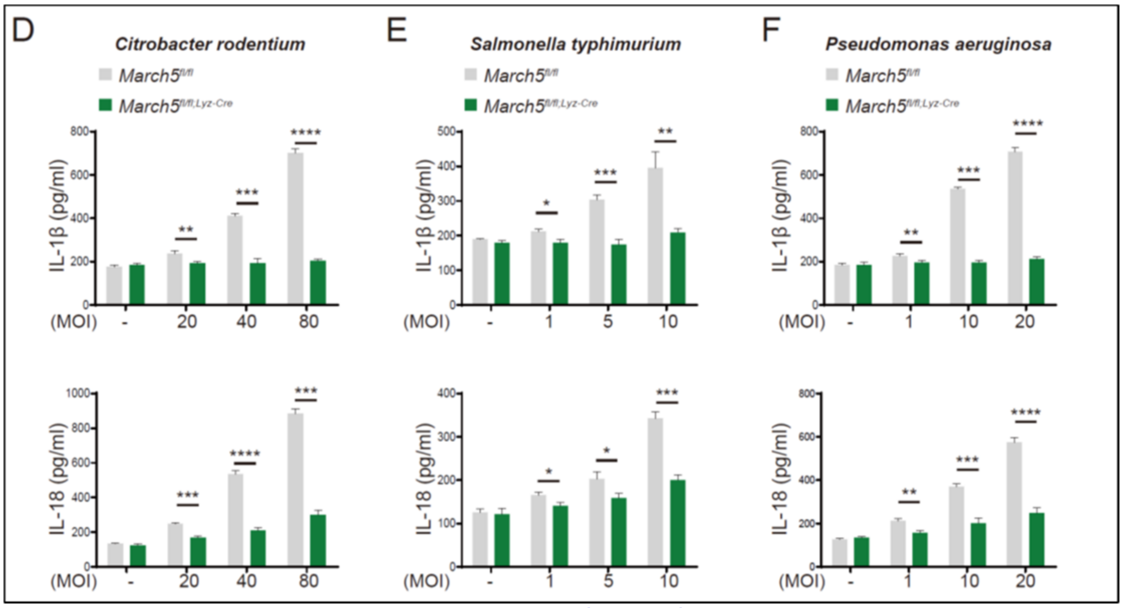

Figure 2D-F (revised)

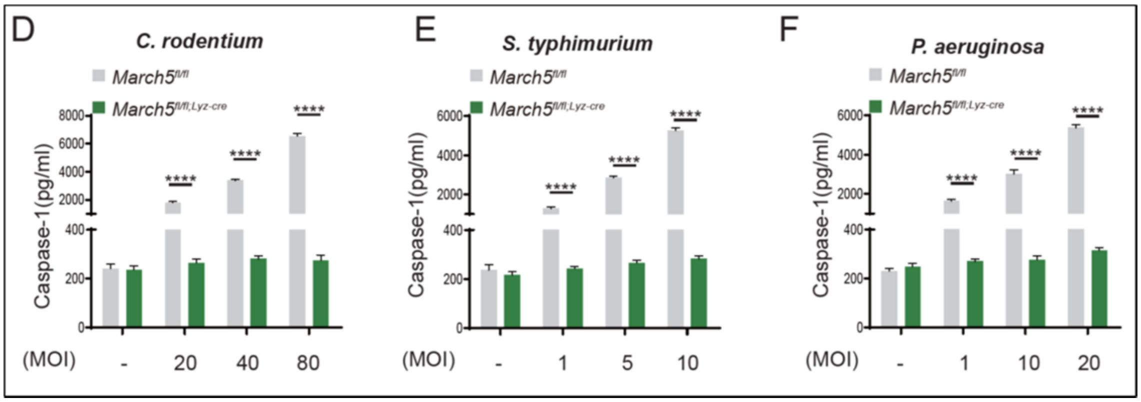

Figure EV3D-F (revised)

Although caspase-1 is an important enzyme for the activation of Pro-IL-18 in macrophages, it is known that mature IL-18 can be also released through a Fas-dependent pathway (Tsutsui H et al., Immunity 1999). Similar to our data, there are other reports showing that IL-18 secretion was observed without Caspase 1 activation in Pellino2 KO BMDMs (Humphries F et al., Nat Commun 2018) or without IL-1β induction (Ren G et al., EMBO J 2019). Thus, it is likely that the gram-negative bacteria used in our study may activate the Fas-dependent pathway or other non-canonical NLRP3 inflammasome. These points were added into the Results (Lines 189-193) of the revised version of manuscript.

References

- Tsutsui H et al. (1999) Caspase-1-Independent, Fas/Fas Ligand–Mediated IL-18 Secretion from Macrophages Causes Acute Liver Injury in Mice. Immunity, 11, 359
- Humphries F, Bergin R, Jackson R, Delagic N, Wang B, Yang S, Dubois AV, Ingram RJ, Moynagh PN (2018) The E3 ubiquitin ligase Pellino2 mediates priming of the NLRP3 inflammasome. Nature communications 9: 1560
- Ren G, Zhang X, Xiao Y, Zhang W, Wang Y, Ma W, Wang X, Song P, Lai L, Chen H (2019) ABRO1 promotes NLRP3 inflammasome activation through regulation of NLRP3 deubiquitination. The EMBO journal 38: e100376

2. In Fig. 4D, the authors conclude that NACHT domain is the binding region because NLRP3-ΔNACHT failed to bind to MARCH5. Is there evidence that NLRP3-ΔNACHT can normally bind to MAVS? If NLRP3-ΔNACHT cannot bind to MAVS, it could simply be because it was not recruited to the mitochondria.

→ Naeha Subramanian et al. (Cell, 2013) reported that only a small part of PYD domain of NLRP3 (aa 2-7) is necessary for its interaction with MAVS. The PYD domain of NLRP3 is intact in the NLRP3-ΔNACHT mutant and thus can bind to MAVS. We also verified that NLRP3-ΔNACHT and NLRP3-ΔLRR containing PYD domain does bind to FLAG-MAVS as shown below.

References

- Subramanian N, Natarajan K, Clatworthy MR, Wang Z, Germain RN (2013) The adaptor MAVS promotes NLRP3 mitochondrial localization and inflammasome activation. Cell 153: 348-361

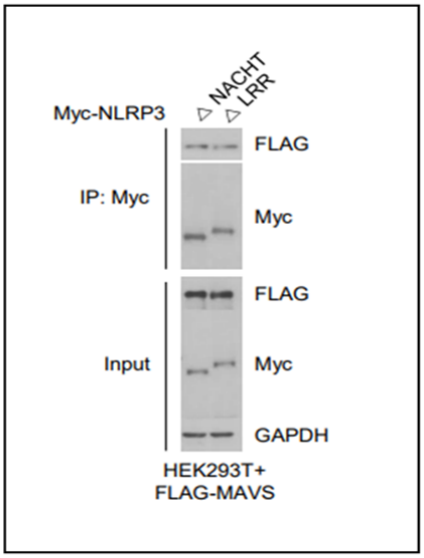

3. In Fig. 5C, KR mutants of Ubiquitin often exhibit unusual behavior. In addition, the K27-dependent ubiquitin chain is less familiar than the K63-dependent ubiquitin chain. To identify Ubiquitin chain types, I recommend additional experiments using deubiquitinating enzymes (UbiCREST Deubiquitinase Enzyme Set, Catalog #: K-400, R&D Systems). This experiment is more convincing than the mutant experiment.

→ We thank the reviewer for your constructive comment. Unfortunately, however, the UbiCREST Deubiquitinase Enzyme Set has been discontinued from the R&D systems as well as from Novus Biologicals. As an alternative, we utilized an OTU deubiquitinase, YOD1, which targets the K6, K11, K27 and K33-linked ubiquitin chains of substrates (Mevissen *et al*, 2013). As a results, we found that YOD1 efficiently removed the ubiquitin chain of NLRP3 induced by MARCH5. The data was added into the Fig. 5E of the revised version and described in Lines 305-309..

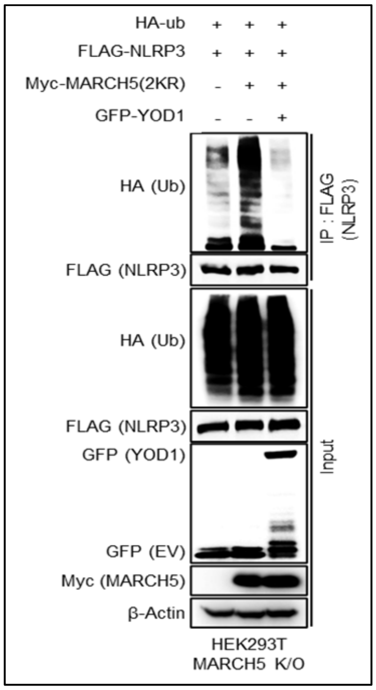

Figure (new data)

4. In relation to Fig. 5E and Fig. 7B,C, why does NLRP3-K93R show a phenotype similar to WT despite reduced ubiquitination? The authors should explain this phenomenon.

Although K93 of NLRP3 is likely to be a target ubiquitination site by MARCH5 (Fig. 5E), ubiquitination-defective mutant of NLRP3 K93R neither interfered ASC oligomerization (Fig. 7B), nor promoted the production of active IL-1β (Fig. 7C). So, MARCH5-mediated ubiquitination at K93 of NLRP3 is not involved in the NLRP3 activation in this study. However, we noticed that NLRP3 K93R mutant rather prompted ASC oligomerization (Fig. 7B).

It is shown that mitochondria-associated NLRP3 oligomers recruits ASC via homotypic PYD-PYD domain interaction during the process of NLRP3 inflammasome assembly (Vajjhala PR et al., JBC 2012; Natarajan S et al. Cell 2013). Thus, we speculate that ubiquitination at NLRP3 K93 next to PYD (aa 1-91) may interfere these interactions, avoiding premature association between NLRP3 and ASC as well as ASC oligomerization before NEK7-NLRP3 binding. These points were added into Discussion (Lines 451-458).

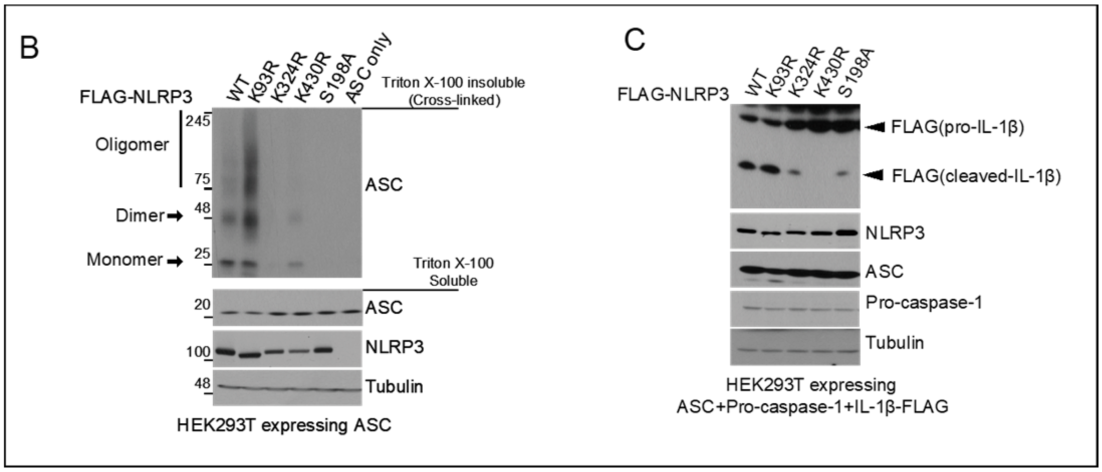

Figure 7 B and C (original data)

References

- Vajjhala PR, Mirams RE, Hill JM (2012) Multiple binding sites on the pyrin domain of ASC protein allow self-association and interaction with NLRP3 protein. J Biol Chem 287:41732

5. The authors claim that ubiquitination of NLRP3 precedes binding of NEK7 to NLRP3.
To prove that the ubiquitination of NLRP3 by MARCH5 is independent of NEK7, it is better to show that NEK7 siRNA.

→We appreciate the reviewer's insightful comment. According to the reviewers' suggestion, a siRNA knocked-down approach for NEK7 was performed. As shown in following results, NEK7 does not involve in NLRP3 ubiquitination by MARCH5 in MARCH5 K/O HEK293T cells.

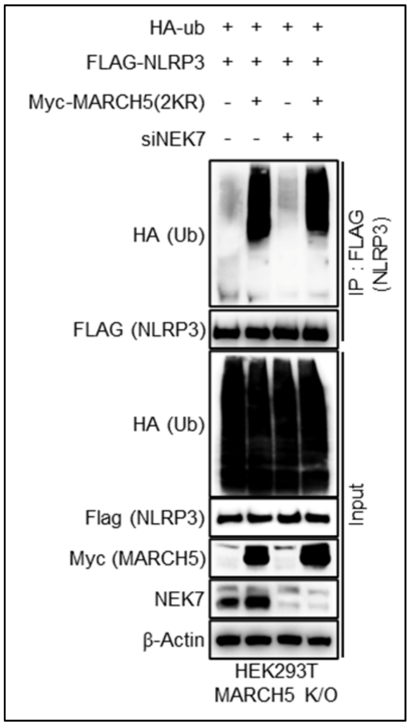

6. The authors argue that phosphorylation of NLRP3 and its translocation to mitochondria occurs prior to ubiquitination of NLRP3 by MARCH5. They should show that ubiquitination of NLRP3-S198A is actually reduced.

→It is shown that in resting macrophages NLRP3 is highly ubiquitinated with mixed Lys-48 and Lys-63 ubiquitin chains. Priming signal by JNK1 induces S194 (S198 in human) phosphorylation, which in turn recruits ABRO1 and BRISC complex that together remove ubiquitin chains (Ren G, et al., EMBO J, 2019). They showed that NLRP3 S198A mutant has more polyubiquitin chain than wild-type NLRP3 upon the NLRP3 activation.

References

- Song N et al. (2017) NLRP3 Phosphorylation Is an Essential Priming Event for Inflammasome Activation, Molecular Cell 68, 185–197
- Ren G, Zhang X, Xiao Y, Zhang W, Wang Y, Ma W, Wang X, Song P, Lai L, Chen H (2019) ABRO1 promotes NLRP3 inflammasome activation through regulation of NLRP3 deubiquitination. The EMBO journal 38: e100376

7. In their previous paper (DOI: 10.1038/ncomms8910), they showed that that Poly:IC stimulation transfers K48-linked polyubiquitin chain on MAVS by MARCH5. Does the stimulation used in this paper promote ubiquitination of MAVS by MARCH5? If not, how do MARCH5 selectively ubiquitinates NLRP3, not MAVs? If ubiquitination does occur, is it possible that the authors have misinterpreted the ubiquitination signal of MAVS bound to NLRP3 as ubiquitination of NLRP3? They should provide evidence to deny this possibility.

→ We thank the reviewer for your thoughtful comment. It was previously reported that Poly:IC stimulation promotes prion-like MAVS oligomers through the interaction with oligomeric RIG-I (Hou F et al., Cell 2011; Peisley A et al., Mol Cell 2013). We found that MARCH5 specifically interacts with

oligomeric MAVS, but not a monomeric MAVS. MARCH5 transfers K48-linked polyubiquitin to the CARD domain of MAVS oligomers as well as of RIG-I oligomers, which led them to a proteasome-dependent degradation. Thus, MARCH5-mediated K48 polyubiquitin on the CARD domain results in protein degradation whereas MARCH5-mediated K27 polyubiquitin on the NACHT domain of NLRP3 generates a signal to allow NEK7 docking. Thus, it is likely that MARCH5 does not transfer the ubiquitin to the CARD domain of MAVS under LPS+ATP/Nigericin stimuli.

References

- Fajian Hou et al. (2011) MAVS forms functional prion-like aggregates to activate and propagate antiviral innate immune response. Cell 146, 448-461
- Peisley A, Wu B, Yao H, Walz T, Hur S. (2013) RIG-I forms signaling-competent filaments in an ATP-dependent, ubiquitin-independent manner. Mol Cell 51,573-583
- Yoo Y et al. (2015) The mitochondrial ubiquitin ligase MARCH5 resolves MAVS aggregates during antiviral signalling. Nat Commun 6,7910
- Park Y-J et al. (2020) Dual targeting of RIG-I and MAVS by MARCH5 mitochondria ubiquitin ligase in innate immunity. Cell Signal 67,109520

(Typos)
1. "This analysis predicted seven ubiquitination sites on Lys (K9, K192, K194, K324, K430, K689, K696) of NLRP3." K93 is correct, not K9

→ We changed K9 to K93.

2. "In this experiment, we found that MARCH5 2KR but not MARCH5 H43W enhanced NLRP3 polyubiquitination independent of K48-linked ubiquitin (Fig S5B)." Fig EV5B is correct, not Fig S5B.
→ We changed Fig S5B to Fig EV5B. We also corrected other typos regarding the same mistakes.

<u>**Referee #2:**</u>

In the manuscript "MARCH5-dependent NLRP3 ubiquitination is an essential step for NEK7 docking on the mitochondria", Park et al. investigate the role of MARCH5, a mitochondrial E3 ligase, in innate immunity. They build this story on in vivo observations on conditional MARCH5 knockout mice with deletion in cells of the myeloid lineage and observe protection in infection and LPS-induced shock models. They then try to connect this to a role of MARCH5 in NLRP3 inflammasome activation. They propose MARCH5 ubiquitinates NLRP3 on the mitochondrial membrane. Upon MAVS binding, this K27-polyubiquitination should trigger NEK7 binding to NLRP3, causing assembly of the NLRP3 inflammasome with the recruitment of adaptor protein ASC and pro-Caspase1. The important role of mitochondria and the ubiquitin system in NLRP3 inflammasome is well establish, yet this is the first report on a role MARCH5 therein. Although the story is potentially interesting, there are some conceptual and experimental concerns.
→ We wish to thank the reviewer for evaluating our work and for allowing us to improve the study. We tried to address the reviewer's concerns and suggestions, and revised the manuscript below.

Major points:
1. The role of MAVS in NLRP3 activation is highly controversial. We would recommend to revisit this and either show a specific involvement here or potentially remove this aspect if not critical to the story.

→ We thank the reviewer for caring thought. Because MARCH5 preferentially binds substrates recruited to the mitochondria, we think that this could be another step-wise regulation in that cytosolic NLRP3 moves to mitochondria. Figure EV6, in particular, showed that MACH5 did not bind NLRP3

and ASC in THP1 cells depleted of MAVS, suggesting that MAVS is involved in the MARCH5 recognition to NLRP3. Ronald Germain's and Emad Alnemri's groups also showed the importance of MAVS in the NLRP3 inflammasome activation.

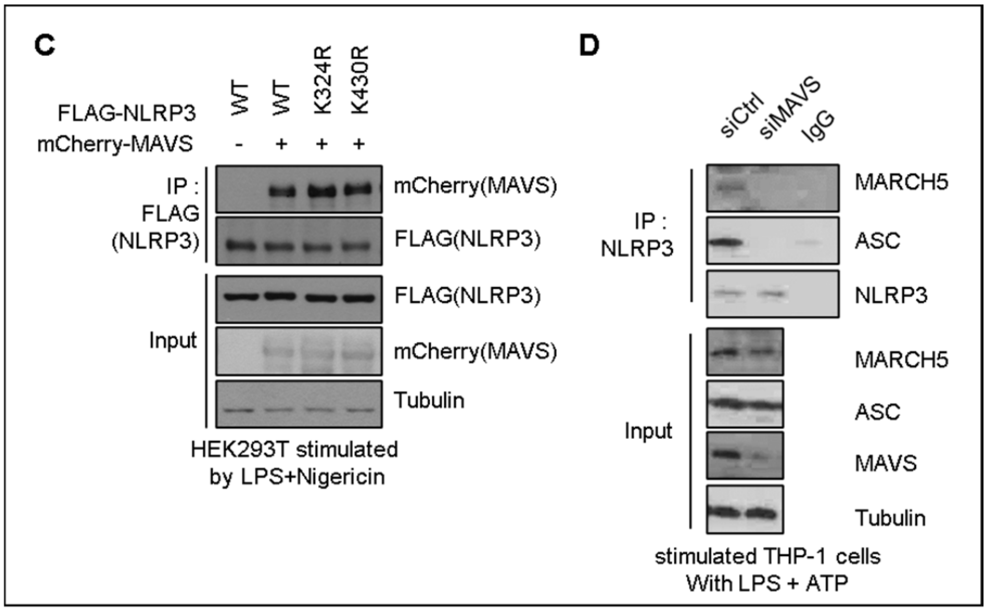

Figure EV6 C and D

References

- Naeha Subramanian, Kannan Natarajan, Menna R. Clatworthy, Ze Wang, and Ronald N. Germain, (2013), The Adaptor MAVS Promotes NLRP3 Mitochondrial Localization and Inflammasome Activation, Cell 153, 348–361
- Sangjun Park, Christine Juliana, Sujeong Hong, Pinaki Datta, Inhwa Hwang, Teresa Fernandes-Alnemri, Je-Wook Yu, and Emad S. Alnemri, (2013), The Mitochondrial Antiviral Protein MAVS Associates with NLRP3 and Regulates Its Inflammasome Activity, J Immunol 2013; 191:4358-4366;

2. NEK7 is not absolutely required for NLRP3 activation. Do the authors think that MARCH5 is also involved in the NEK7-independent mechanism of NLRP3 activation?

→ As mentioned by the reviewer, IKKβ-mediated NEK7-independent NLRP3 pathway on the trans-Golgi network is shown by Hornung's group (Immunity 2022). Among 11 members of MARCH family, MARCH5 is specifically present on the mitochondrial outer membrane, but not on the Golgi apparatus. Thus, it is less likely that MARCH5 functions in the NEK7-independent NLRP3 activation in the Golgi apparatus. At the conditions of NEK7 knockdown by siRNA, MARCH5 still induced the NLRP3 ubiquitylation as shown below, suggesting that MARCH5-mediated NLRP3 ubiquitination occurs regardless of NEK7.

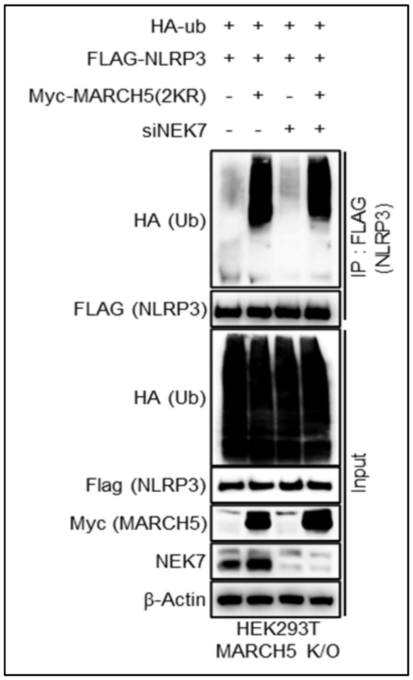

References

- Mann M et al. (2022) IKKβ primes inflammasome formation by recruiting NLRP3 to the trans-Golgi network. Immunity 55, 2271.

3. At some points, e.g., Fig 2., a direct comparison to NLRP3 knockout cells should be included to estimate the degree of MARCH5-dependency.

→ As the reviewer mentioned, NEK7-independnt NLRP3 activation can occur. Moreover, it is not addressed whether MARCH5 regulates NLRP11-mediated caspase-1 activation or Fas-dependent IL-18 secretion. Thus, we assumed that MARCH5 knockout may not always follow the phenotype of NLRP3 knockout.

References

- Tsutsui H et al. (1999) Caspase-1-Independent, Fas/Fas Ligand–Mediated IL-18 Secretion from Macrophages Causes Acute Liver Injury in Mice. Immunity, 11, 359
- Gangopadhyay A. et al. (2022) NLRP3 licenses NLRP11 for inflammasome activation in human macrophages. Nature Immunology 23, 892–903

4. Salmonella is not a typical NLRP3 activator and also triggers other inflammasomes. Do the authors think that MARCH5 could also impact other inflammasomes and how would this relate to mitochondria?

→ As mentioned by the reviewer, *Salmonella* is known to activate NLRP3 and NLRC4 (Broz *et al*, 2010; Man *et al*, 2014). Our inflammation assay data using MARCH5 KO and WT BMDMs after treatment with various stimuli (Figures 2A-2C) revealed that MARCH is likely only involving in the NLRP3 activation upon *Salmonella* stimuli. In the revised version, we also determined the released caspase-1 levels upon various stimuli in addition to IL-1β and LDH levels.

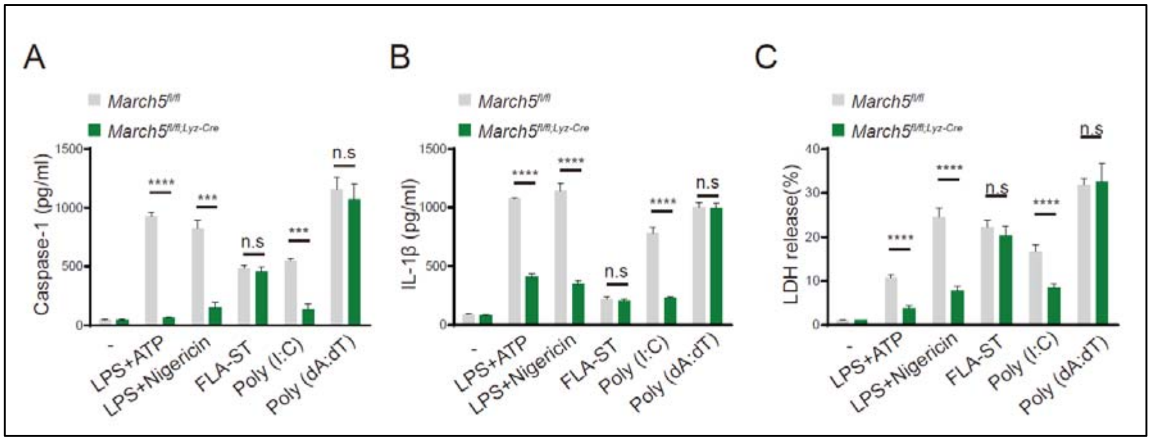

Figures 2A-2C

→MARCH5 has no effect on NLRC4 (FLA-ST) and AIM2 (poly (dA:dT))-mediated pathways. Recently, poly (dA:dT) was also found to activate NLRP1 (Zhou *et al*, 2023). Poly (I:C) is known to stimulate NLRP1 and NLRP3 pathways while LPS+ATP/Nigericin stimulates the NLRP3 inflammasome. Together, it is likely that MARCH5 is only involved in the NLRP3 activation upon *Salmonella* stimuli. MARCH5 localizes the outer membrane of mitochondria and cytoplasmic NLRP3 moves to mitochondria for its activation as shown in our model (Fig. 7E).

References

- Broz P, Newton K, Lamkanfi M, Mariathasan S, Dixit VM, Monack DM (2010) Redundant roles for inflammasome receptors NLRP3 and NLRC4 in host defense against Salmonella. Journal of Experimental Medicine 207: 1745-1755
- Man SM et al. (2014) Inflammasome activation causes dual recruitment of NLRC4 and NLRP3 to the same macromolecular complex. Proceedings of the National Academy of Sciences 111: 7403
- Zhou JY, Sarkar MK, Okamura K, Harris JE, Gudjonsson JE, Fitzgerald KA (2023) Activation of the NLRP1 inflammasome in human keratinocytes by the dsDNA mimetic poly (dA: dT). Proceedings of the National Academy of Sciences 120: e2213777120

5. Throughout the paper, inflammasome activation seems to be suboptimal with low IL-1β or LDH values and a low cell frequency of inflammasome activation. This not only raises doubts about the experimental prowess, but also the concern that the phenotype is only observed under suboptimal stimulation conditions but lost under optimal conditions. Dose/response and time-course experiments that reach the plateau should be shown, e.g., with nigericin. (LPS and nigericin concentrations used throughout the article are relatively high, maybe too high). Frequency as in Fig. 7 should also be included in earlier data sets such as in Fig. 2. FACS data for pyroptosis could also be useful is this respect.

→ We thank the reviewer for thoughtful comment. We had chosen the dose and time for LPS+Nigericin/ATP treatment after time-course experiments. As shown below, we found that the cleaved IL-1 β appeared at the concentration of 15 uM of Nigericin but not of 7.5 uM. We also chose 30-60 min where the cleaved IL-1β levels was apparently elevated.

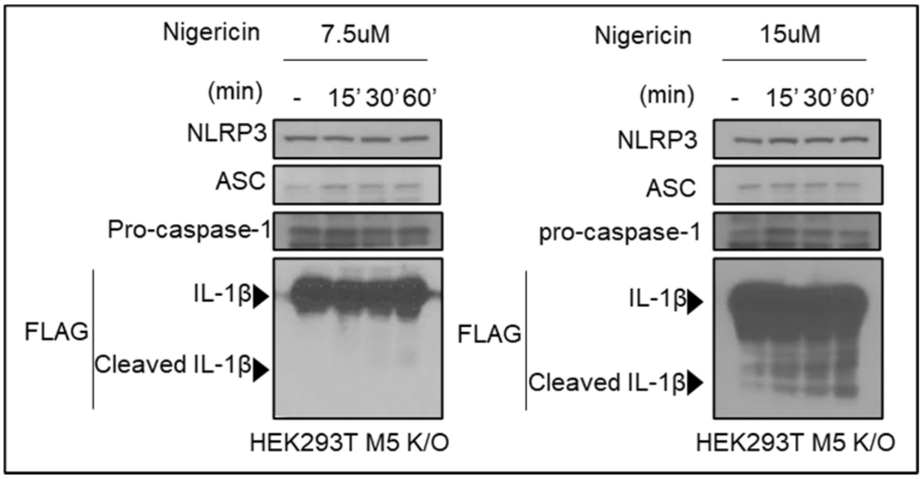

6. Why are different MOI used for different read-outs for the three bacterial strains, as in Fig 2C? The IL-18 levels detected here are quite high, while the concentrations of the other cytokines measured here are very low.

→ We appreciate the reviewer for finding our mistake in the Fig.2C &D of which read-outs of IL-1β were switched. In the revised manuscript, we repeated the assay and corrected them as shown below.

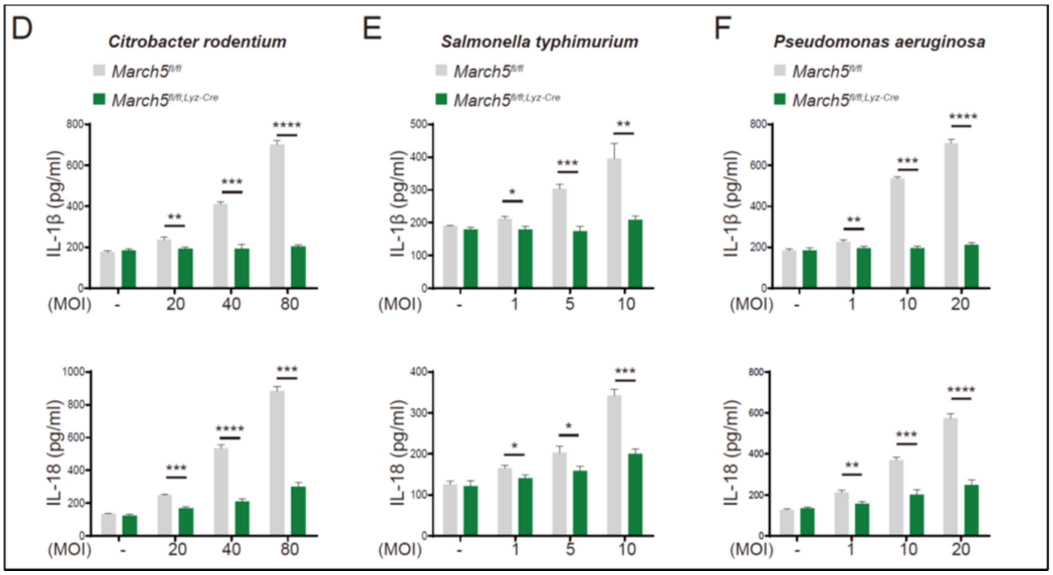

Figure 2D-F (revised)

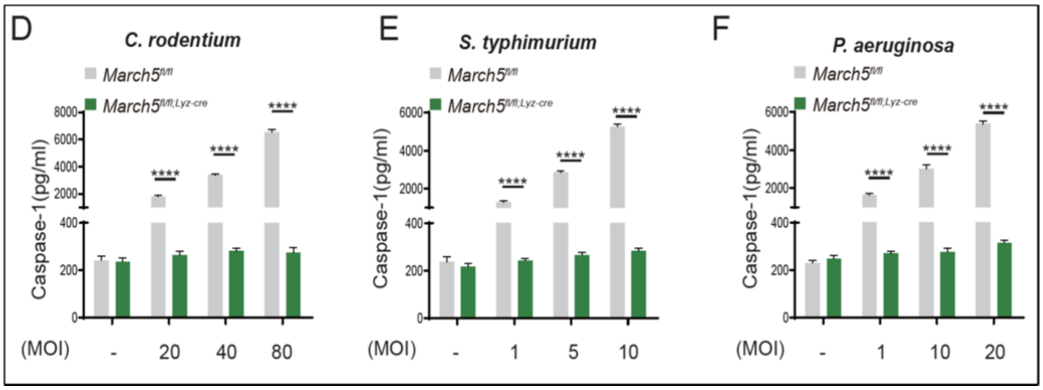

Figure EV3D-F (revised)

→We experienced that those three bacterial strains resulted in different levels of cytokine production. So, we chose different doses of MOIs to have similar amounts of cytokine production. After correcting our mistakes, the concentrations of cytokine production appeared to be similar. In addition, IL-18 can be also released through a Fas-dependent pathway (Tsutsui H et al., Immunity). Thus, it is likely that the gram-negative bacteria used in our study may activate the Fas-dependent pathway or other non-canonical NLRP3 inflammasome. These points were added into the Results (Lines 189-193) of the revised version of manuscript.

References

- Tsutsui H et al. (1999) Caspase-1-Independent, Fas/Fas Ligand–Mediated IL-18 Secretion from Macrophages Causes Acute Liver Injury in Mice. Immunity, 11, 359

7. Why is the MARCH5 phenotype seen for nigericin and ATP much less clear than for live pathogens? We would expect the opposite.

→We thank the reviewer for precise comment. We again carried out the experiment using a higher number of BMDMs isolated from *March5*$^{fl/fl}$ and *March5*$^{fl/fl;Lyz\text{-}Cre}$ mice. We observed increased levels of caspase-1, IL-1β and LDH release. The revised data was placed into Fig 2A-C.

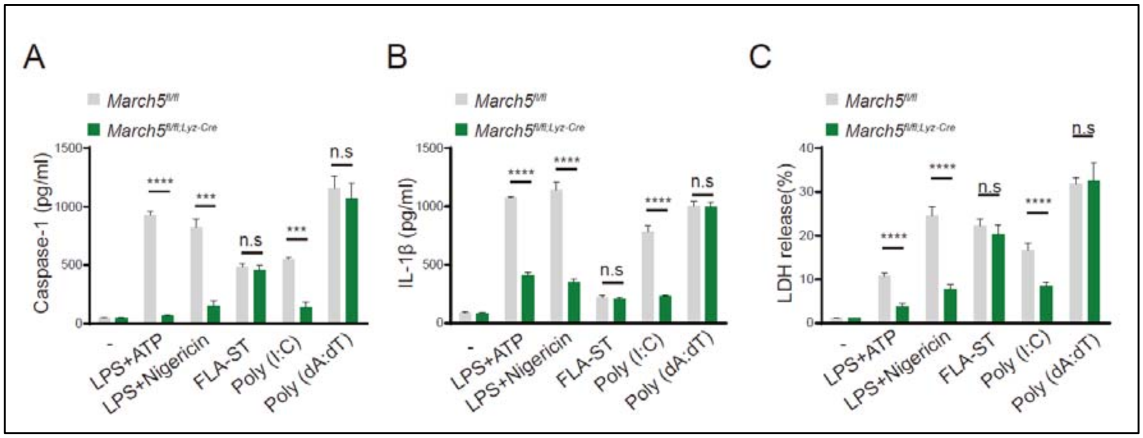

Figures 2A and 2B (revised)

8. UbPred predicted only 7 ubiquitination sites on NLRP3 while there are more sites reported in the literature already. Why did the others not show up in the prediction and why were not considered in the experimental analysis? Could a proteomics approach have been used to determine Ub sites?

→In the analysis of UbPred prediction, they also showed the different score levels. We selected sites with high confident score.

9. At the end of the discussion, the authors propose to regulate MARCH5 activity as a new therapeutic strategy. Potential side effects should be discussed. What is the phenotype of MARCH5 full knockouts? What phenotype could be expected when deleting MARCH5 in adults, e.g., using ROSA26-ERT2-Cre mice? What effect would global inhibition of MARCH5 have in mice and humans? How likely is it to be able to develop a strategy that only affects the effect of MARCH5 on NLRP3 but not its other functions

→ Mice with *March5* full knockout are embryonic lethal. In addition to NLRP3 activation, MARCH5/MITOL shows a variety of cellular functions as discussed in the review by Yanagi Shigeru (2020). Accordingly, we think that proteolysis-targeting chimera (PROTAC) approach specifically linked to NLRP3 would be the way.

References

· Shiiba I. et al (2020) Overview of Mitochondrial E3 Ubiquitin Ligase MITOL/MARCH5 from Molecular Mechanisms to Diseases. 27;21(11):3781.

10. Westerns blots for Casp1 activation and to exclude effects on pro-IL-1β levels are missing throughout. Data in Fig 3A suggests an effect of MARCH5-deficiency on NLRP3 expression. This should be verified or excluded.

→ We repeated the experiment of Fig. 3A and replaced it in the revised manuscript. In Fig. 3B, we also determined the caspase-1 activity in BMDMS.

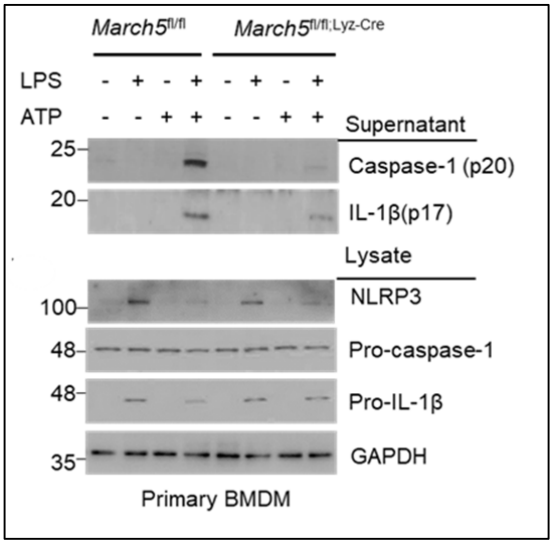

Figures 3A (revised)

11. The THP-1 data in Fig EV4A is not convincing, cleaved Casp1 bands are blurry, and the IL-1β band in the knockout seems unaltered. THP-1 cells should express NLRP3 and pro-IL-1β without LPS due to differentiation with PMA. There is a typo also in this panel. Nigericin might work better than ATP in THP-1 cells.

→ We repeated the experiment of Fig EV4A and replaced it in the revised manuscript. We thank the reviewer for pointing out our mistake and corrected it.

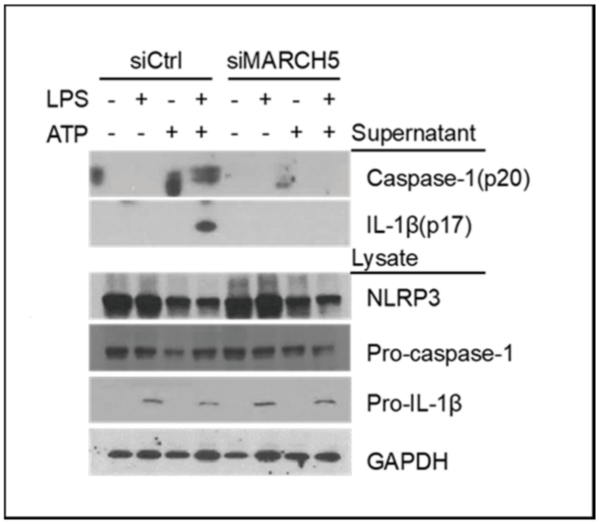

Figure EV4A(revised)

Specific points:

12. Fig 3B: in the casp1 activity assay, RLU levels are really low. Was it tested with other activators? Is there data for THP-1 cells?

→ According to the reviewers' comment, we repeated the experiment. In this time, we evaluated Caspase-1 secretion using ELISA after seeding a higher number of BMDMs isolated from *March5*$^{fl/fl}$ and *March5*$^{fl/fl;Lyz\text{-}Cre}$ mice and found a significant increase in Caspase-1 level. These results were placed in revised Figure 3B. Additionally, we tested the secretion of Caspase-1 upon treatment with various inflammasome activators (shown in B) and added into the revised Figure 2A.

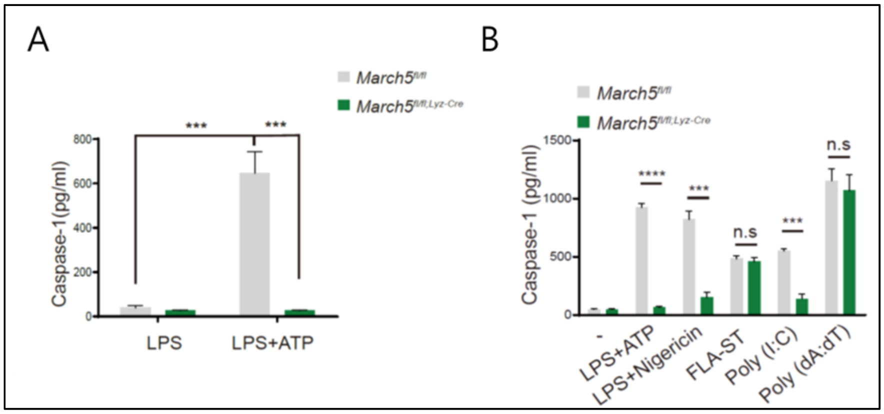

We also performed the same experiments in THP-1 cells. First, we prepared the MARCH5 knockdown THP-1 cells using MARCH5 specific siRNA and measured the levels of caspase-1, IL-1β and LDH release upon treatment of various inflammasome activators. We found similar results to those obtained from MARCH5 BMDM cells. The data was added into EV3 A-C of the revised version.

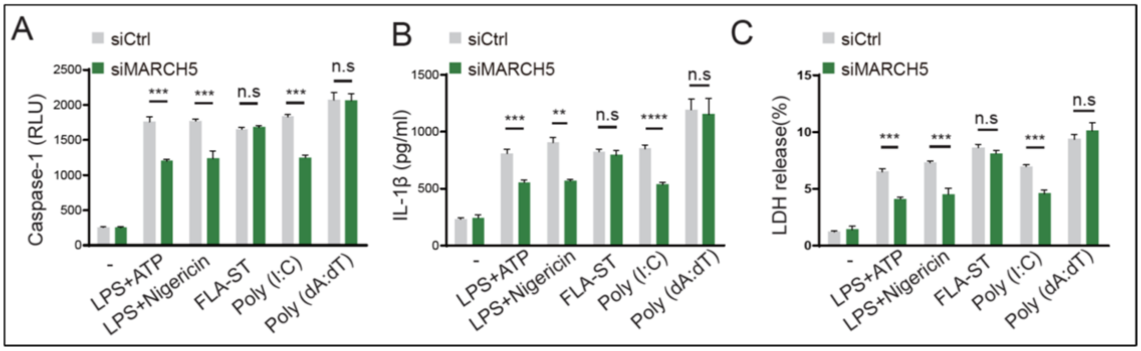

13. Fig 3: C,D: which time points were used for ATP and Nigericin treatment? 45 min as for ATP treatment in Fig 3A?

→ We used 45 min for ATP treatment and 60 min for Nigericin treatment. We added time points in the figure legend of the revised version.

14. Fig 3D, why is there an ASC dimer background in the untreated condition?

→ To detect ASC oligomerization, we treated DSS for cross-linking. It may cause the background also shown in other paper (Cell 2013;153, 348).

15. Fig 3E, why such a low frequency with nigericin or ATP? Were cells transfected with Poly(dA:dT) not primed with LPS?

→ We thought that MARCH5-mediated ubiquitination occurs early step during the process of NLRP3 inflammasome assembly and activation. So, the frequency of ASC specks might be low under these conditions (15 min, Nigericin/ATP) compare to others (45-60 min). We did not co-treat LPS in the case of Poly(dA:dT).

16. Fig 4D, we see a band there for deltaNACHT.

→ We repeated the experiment and replaced it as shown below.

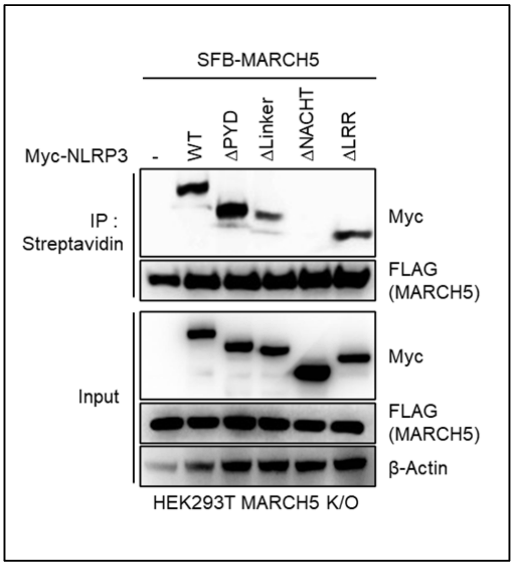

Figure 4D(revised)

17. Fig 5D, the labelling of the X-axis is difficult to understand. The figure legend should outline in more detail what the labelling is referring it. There are no error bars for the condition MARCH KO HEK293T expressing K27O and transfected with Myc-MARCH5 H43W mutant? Prism settings? Nomenclature with K27O and K27R is misleading. K27R is never explained (only K48R previously).

→ We added the error bars in MARCH KO cells and described the nomenclature of K27O and K27R in the Result (Lines 300-302); K27O ub contains only one intact Lys residue at K27, and the other six Lys residues were mutated to Arg whereas K27R ub contains Arg residue at K27 and the other six Lys residues are intact.

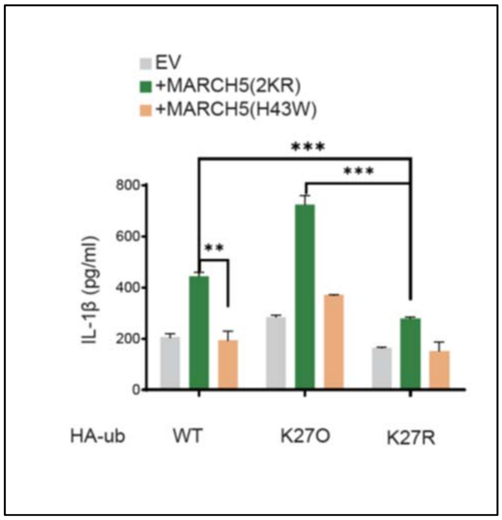

Figure 5D(revised)

18. Fig 5E, what about K93R, why was this not followed up? How can mutation of two individual sites each lead to an almost complete loss in ubiquitination? There is a typo on predicted Ub Sites: K189 is written instead of K689 on the graph.

→ Although K93 of NLRP3 is likely to be a target ubiquitination site by MARCH5 (Fig. 5E), ubiquitination-defective mutant of NLRP3 K93R neither interfered ASC oligomerization (Fig. 7B), nor promoted the production of active IL-1β (Fig. 7C). So, MARCH5-mediated ubiquitination at K93 of NLRP3 is not involved in the NLRP3 activation in this study. We corrected the typo of K189R to K689R on the graph.

19. In the first part of the results where MARCH5 immune-modulatory functions are investigated, the authors claim no significant difference between TNF and IL-6 levels in P.aeruginosa infected mice and control group. In the endotoxemia-induced septic shock model, they then say that the results were "inconsistent" with the previous model. However, they come to the same conclusion of no difference between TNF and IL-6 levels. Please check or specify what is meant with "inconsistent" here.

→We made the mistake and changed to “consistent”.

20. Typo in "MARCH5 promotes NLRP3 activation via K27-linked polyubiquitination of the Lys324 and Lys430 residues of NLRP3" paragraph: figure EV5B is referred to as S5B.

→We changed to “MARCH5 transfers K27-linked polyubiquitin chains to the K324 and K430 residues of NLRP3”. We also changed S5B to EV5B.

21. Fig EV5E: why is there no pro-IL-1β in the blot shown in cell lysate or IL-1β p17 in supernatant to confirm ELISA results?

→We added the pro-IL-1β levels in Fig EV5E.

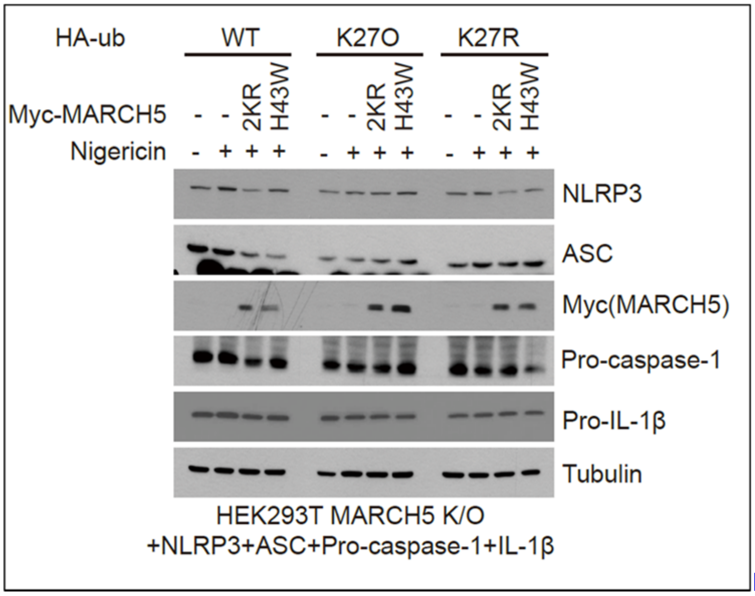

Figure EV5E 5D(revised)

22. In the discussion: typo at the sentence "Thus, the data suggest that NLRR3 recruitment to mitochondria by MAVS precedes its interaction with MARCH5" NLRR3 instead of NLRP3.
→We changed to "NLRP3' in that sentence.

23. In the discussion: "We observed that cells expressing the NLRP3-S194A mutant also failed to form the multimeric NEK7-NLRP3 complex (Figs 6E and F)." However, experiments were made in human cells (S198A mutant) and not in mouse.
→We changed to "NLRP3-S198A mutant" in that sentence.

24. Material section missing reference for anti-HA antibody.
→ We added the resources for anti-HA antibody.

25. The figure legends are inconsistent at times (e.g., time points missing)
→ We described the figure legend more detail.

Referee #3:

Mitochondria are known to be intensely involved in inflammasome activation as they increase the production of reactive oxygen species (ROS) and release oxidized fragments of mitochondrial DNA into the cytosol, which stimulates inflammasome activation. The manuscript by Park et al. now describes that the human inflammasomal protein NLRP3 is ubiquitinated by the mitochondria-associated E3-ligase MARCH5. Using truncation and mutagenesis studies the authors show that MARCH5 interacts with the NACHT domain of NLRP3 and promotes K27-linked polyubiquitination of K324 and K430 residues of NLRP3. MARCH5 knockout mice failed to secrete IL-1b and IL-18 from macrophages upon bacterial infection and exhibited an attenuated mortality rate. Ubiquitination at the two sites in the NACHT domain is required for NEK7 binding and NLRP3 oligomerization, suggesting that the E3 ligase MARCH5 is an important regulator of NLRP3 function.

Overall, the authors present a comprehensive study, starting with an analysis of the E3 ligase in bacterial activation assays (Figs 1, 2), the assignment to the activation mechanism of NLRP3 (Fig. 3), a mapping to the NACHT domain of NLRP3 (Fig. 4), the molecular characterization of K27-ubiquitination and identification of two (three) sites on the target protein (Fig. 5), the requirement of NLRP3 ubiquitination for NEK7 binding and activation on a molecular level (Fig. 6) and on a functional

level, analysing IL-1beta release (Fig. 7).

The experimental part seems thoroughly performed, but the text is rather heterogeneously written. While the Abstract and the Introduction sections need serious editing, the Results and Discussion sections are well written. I have a couple of comments to the present version of the manuscript.

→ We wish to thank the reviewer for evaluating our work and for allowing us to improve the study. We tried to address the reviewer's suggestions, and revised the manuscript. In addition, we rewrote parts of Abstract and Introduction.

Comments:

In the cellular ubiquitination assay using K-to-R mutants the signal loss for the K93R mutant appears as strong as for the K430R mutant, and indeed much more than for the K324R mutant. It is well taken that the E3 ligase many interact with the NACHT domain, but why did the authors omit the K93 site in the following? Interestingly, the K93R mutant shows even stronger ASC dimer and oligomerization bands in Figure 7B. Could this be a negative regulation, such that K93-Ub prevents inflammasome formation, while K430-Ub stimulates it?

→ We thank the reviewer for thoughtful comments. Although K93 of NLRP3 is likely to be a target ubiquitination site by MARCH5 (Fig. 5E), ubiquitination-defective mutant of NLRP3 K93R neither interfered ASC oligomerization (Fig. 7B), nor promoted the production of active IL-1β (Fig. 7C). So, MARCH5-mediated ubiquitination at K93 of NLRP3 is not involved in the NLRP3 activation in this study. However, we noticed that NLRP3 K93R mutant rather prompted ASC oligomerization (Fig. 7B).

It is reported that mitochondria-associated NLRP3 oligomers recruits ASC via homotypic PYD-PYD domain interaction during the process of NLRP3 inflammasome assembly (Vajjhala PR et al., JBC 2012; Natarajan S et al. Cell 2013). Thus, it could be a negative regulation and we may postulate that ubiquitination at NLRP3 K93 next to PYD (aa 1-91) may interfere these interactions, avoiding premature association between NLRP3 and ASC as well as ASC oligomerization before NEK7-NLRP3 binding. These points were added into Discussion (Lines 451-458).

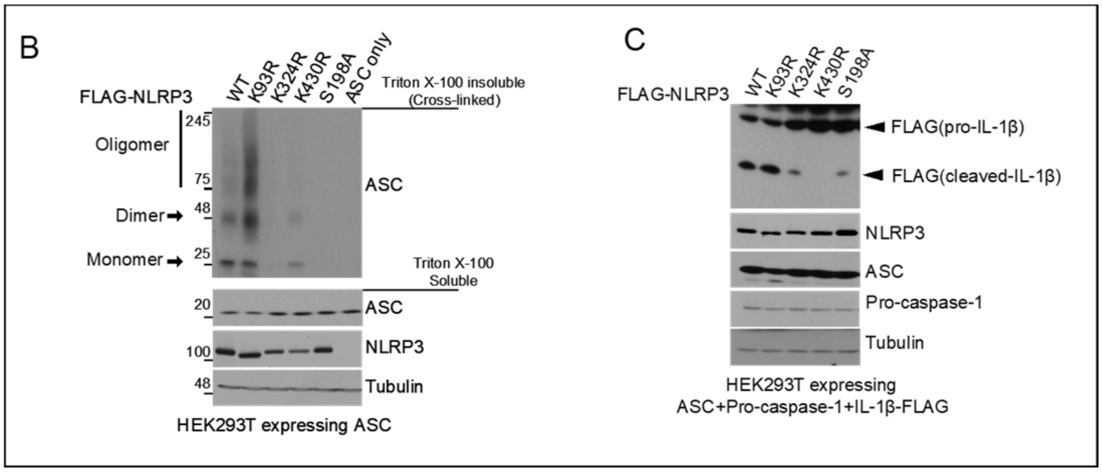

Figure 7 B and C (original data)

References

- Vajjhala PR, Mirams RE, Hill JM (2012) Multiple binding sites on the pyrin domain of ASC protein allow self-association and interaction with NLRP3 protein. J Biol Chem 287:41732
- Subramanian N, Natarajan K, Clatworthy MR, Wang Z, Germain RN (2013) The adaptor MAVS promotes NLRP3 mitochondrial localization and inflammasome activation. Cell 153: 348-361

How do the identified ubiquitination sites fit to the recent structural data of the active NLRP3-NEK7 inflammasome (Xiao et al., 2023)? Is there a molecular rational for the diminished NEK7 binding of the K324 and K430 arginine mutants (Fig. 6E)? And how do the sites align to the resting state of NLRP3 (Andreeva et al, Hochheiser et al)? Could ubiquitination lead to an opening of the NACHT domain, as described in the Discussion section as "rotational activation" (page 13, last paragraph).
→Again, we thank the reviewer for thoughtful comments.

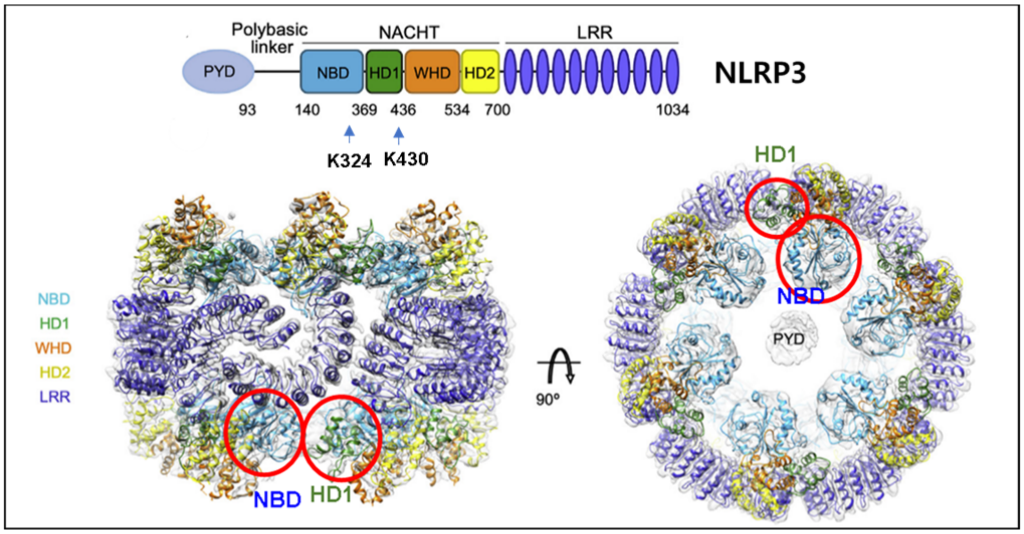

NEK7 interacts with mainly LRR domain and a part of NACHT domain (WHD and HD2). MARCH5-mediated NLRP3 ubiquitination occurs at the NBD and HD1 subdomains of NACHT neighboring to NEK7-NLRP3 interaction sites. In the resting state of NLRP3 (Andreeva et al), NDB and HD1 subdomains are shown above.

It is suggested that the central NACHT domain of NLRP3 oligomers/cages rotates upon ATP binding and then binds to NEK7(Xiao et al.). NEK7 binding to NLRP3 cage opens the cage into two halves that are finally converted to active ring-like shape of NEK7-NLRP3 complex. We postulate that MARCH5-mediated ubiquitination on NLRP3 may contribute to the rotational activation to form the ring-like shape of NLRP3-NEK7 oligomers. It was proposed that 90° rotation of NBD and HD1 (MARCH5-mediated ubiquitination sites) may occur before NEK7 binding (Sharma M. et al) as we mentioned in the Discussion section.

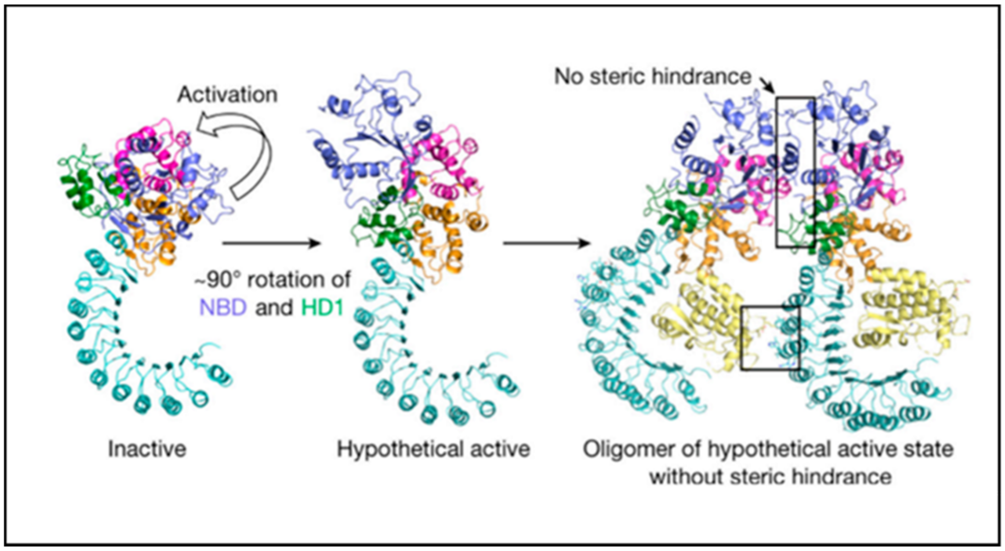

In addition, ubiquitin E3 ligases transfer ubiquitin to Lys residue of target proteins. Thus, K324 and K430 arginine mutants are not ubiquitylated by MARCH5, showing the diminished NEK7 binding.

I would like to mention that structural modeling on NEK7-NLRP3 (NEK7-NLRP3-ASC complex) is largely based on NLRP3 complexes located on the Golgi Apparatus. Recent report (Immunity, 2022) by Veit Hornung's group showed the NEK7-independent NLRP3 activation on the Golgi Apparatus.

References

- Schmacke NA et al. (2022) IKKβ primes inflammasome formation by recruiting NLRP3 to the trans-Golgi network. Immunity 55:2271
- Sharma, M.; de Alba, E.(2021) Structure, Activation and Regulation of NLRP3 and AIM2 Inflammasomes. Int. J. Mol. Sci. 22, 872.

Minor:
Page number (or line numbers) are missing, which is bad.
→ We apologize the reviewer for this inconvenience. We added pages and line numbers in the revised version.

Introduction, page 4, second line: "In the resting state, inactive NLRP3 retails its folded structure, but it converts to the unfolded form ..." Where has this been shown? Please give a citation for this statement. It now rather appears, that inactive NLRP3 is an oligomer (decamer, PDB ID: 7pzc) as is the active NLRP3-NEK7 complex (hetero-decamer, PDB ID: 8ej4).

→ According to Roman Jerala's group (Nat Comm, 2013), cytosolic monomeric NLRP3 forms the folded PYD structure interacting with NACHT domain and these inhibitory interactions are released upon activation, which allows forming oligomeric NLRP3. As far as we understand, this oligomeric NLRP3 (decamer) is still inactive before binding to NEK7. To avoid the confusion, we wrote to "In the resting state, NLRP3 retains its folded structure hiding pyrin domain, but it converts to the oligomers

during activation" in the revised version.

References

- Hafner-Bratkovič I et. A. (2018). NLRP3 lacking the leucine-rich repeat domain can be fully activated via the canonical inflammasome pathway. Nat Comm 9, 5182

Figure 4A and 4B: the lanes are not well labelled, see, e.g. the "-" and "+" in panel B. Same holds for Fig. 5E.
→ Labeling on Fig. 4A, 4B and 5E was corrected in the revised version.

Discussion: "NLRP3 phosphorylation at Ser194 is a key priming event ..." Please use human NLRP3 numbering throughout. This site was designated as S198 elsewhere in the manuscript (Fig. 7, ...).

→ We corrected to "NLRP3 phosphorylation at Ser198 is a key priming event". We also changed to S198 throughout figures and text.

I hope that we now fulfil the requirement on our manuscript. We look forward to hearing from you regarding your final decision.

Yours sincerely,

Hyeseong Cho, PhD

Professor Emeritus, Department of Biochemistry and Molecular Biology

Ajou University School of Medicine

World cup-ro, Yeongtong-gu, Suwon 16499, Korea

Tel.: +82-31-219-5052, E-mail: hscho@ajou.ac.kr

1st Revision - Editorial Decision 18th Jul 2023

Dear Hyeseong,

Thank you for submitting your revised manuscript to The EMBO Journal. I am sorry for the delay in getting back to you with a decision, but your study has now been seen by the original referees and their comments are provided below.

As you can see, the referees appreciate the introduced changes and support publication here. Referee #2 has a few remaining suggestions that I would like to ask you to take into consideration. I think that you have the data on hand to address the issues. If not let's discuss.

Also, take a look at 6C it looks like it has some grid-like marks in the figure panel. I see the marks also in the source data and I don't have an issue with them, but just want to point them out to you.

Let me know if we need to discuss anything further

With best wishes

Karin

Karin Dumstrei, PhD
Senior Editor
The EMBO Journal

Instructions for preparing your revised manuscript:

Guide For Authors: https://www.embopress.org/page/journal/14602075/authorguide

----

Referee #1:

Our comments are adequately answered in the revised version. I recommend that this paper be accepted for publication in EMBO J.

Referee #2:

We have now looked at the revised version and the authors' replies. We still think that a role for MARCH5 in NLRP3 activation is an interesting and potentially important observation, as the in vivo data implies.

The authors answered most of our questions experimentally, but not all:

Point #3: Analysis on BMDMs from NLRP3 knock-out mice.

Point #5: Time course and dose-response data.

If the authors have time course and dose-response data that reaches a plateau directly comparing wild-type and MARCH5 knockout cells, that would in a way also address my NLRP3 question and I would not demand that they get knockout bone marrow at this point.

The issue concerning the (controversial) role of MAVS and NEK7 in NLRP3 activation is not properly reflected. The authors should considered a more nuanced tone regarding this.

Referee #3:

This is a detailed revision of the manuscript by Park and co-workers describing the mitochondria-associated E3-ligase MARCH5 as a ubiquitination enzym of the NLRP3 protein. The comments from all three reviewers are well addressed by additional experiments and changes made, as I may judge from my perspective/ knowledge. As this is a timely study on the regulation of NLRP3, I am in favour of publication of this manuscript.

Karin Dumstrei, PhD
Senior Editor
The EMBO Journal

July 20, 2023

Manuscript ID: EMBOJ-2023-113481R

Manuscript Title: MARCH5-dependent NLRP3 ubiquitination is an essential step for NEK7 docking on the mitochondria

Dear Karin,

We thank you again for your time and consideration on our submission. Below we addressed the Referee #2's and your comments.

In response to Referee #2, we provided our previous data determining IL-1β concentration in LPS plus Nigericin treated BMDMs derived from *March* WT and KO mice. Regarding the issue on MAVS, we changed the text by simply describing the results of the figures (Appendix Fig S1C, D) with a more nuanced tone we think. Thirdly, we corrected 6C by removing marks.

I will greatly appreciate if you can give me the decision at your earliest convenience.

With my best regards,

Hyeseong

Hyeseong Cho
Ajou University School of Medicine
Suwon, Korea

………………………………………………………………………………..

Responses to the Reviewers' comments (comments from reviewers in **black,** responses to the reviewers in **blue)**

Referee #2:

We have now looked at the revised version and the authors' replies. We still think that a role for MARCH5 in NLRP3 activation is an interesting and potentially important observation, as the in vivo data implies.

The authors answered most of our questions experimentally, but not all:

Point #3: Analysis on BMDMs from NLRP3 knock-out mice.

Point #5: Time course and dose-response data.

If the authors have time course and dose-response data that reaches a plateau directly comparing wild-type and MARCH5 knockout cells, that would in a way also address my NLRP3 question and I would not demand that they get knockout bone marrow at this point.

→ We thank the reviewer for constructive comment. In our previous screening, we had chosen 15 μM of Nigericin after determination of the IL-1β level in LPS-primed BMDMs derived from *March5*$^{fl/fl}$ and *March5*$^{fl/fl;Lyz\text{-}cre}$ mice as shown below. The IL-1β secretion was the highest with 15 μM of Nigericin treatment for 30 min in LPS-primed *March5*$^{fl/fl}$ BMDMs while the IL-1β secretion in LPS-primed *March5*$^{fl/fl;Lyz\text{-}cre}$ BMDMs reaches a plateau after 10 μM of Nigericin treatment. In the literatures, 2.5-5 mM of ATP and 7.5-15 μM of Nigericin concentrations are widely used and however, we noticed relatively low IL-1β levels in our data as the reviewer pointed out. When we discussed these points in our lab, we think that differences may come from different ELISA systems. There are two types of ELISA kit commercially available. One is provided with antibody-coated plate (expensive) and the other is given with antibody vials that we have used.

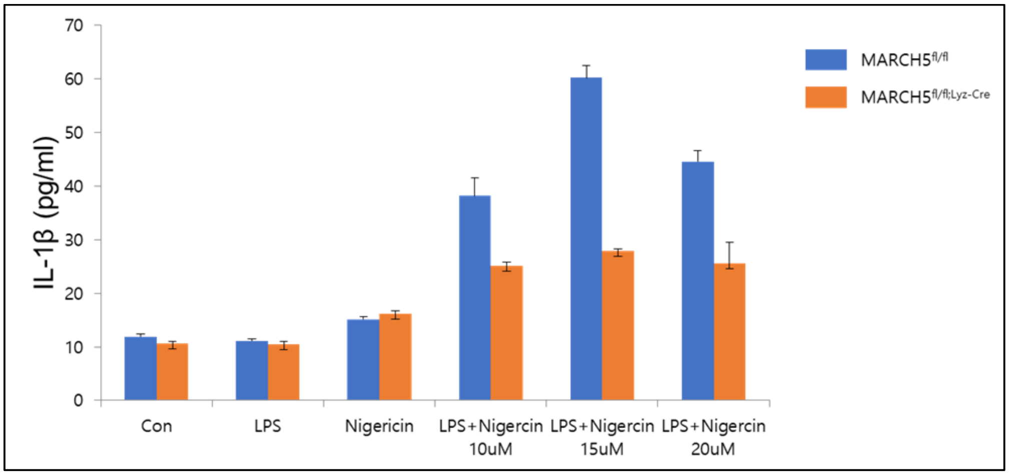

The issue concerning the (controversial) role of MAVS and NEK7 in NLRP3 activation is not properly reflected. The authors should considered a more nuanced tone regarding this.

→ According to reviewer's suggestion, we changed the text of Results by simply describing the data with a more nuanced tone we think (Lines 340-351). "It was previously shown that NLRP3 interacts with mitochondrial lipid cardiolipin, MFN2 and MAVS, which contributes to the NLRP3 inflammasome activation (Park *et al*, 2013; Iyer *et al*, 2013; Subramanian *et al.*, 2013). It also shows that NEK7 directly binds NLRP3, and these interactions are essential for NLRP3 oligomerization (He *et al*, 2016; Sharif *et al.*, 2019). As shown in Appendix Fig S1B, coimmunoprecipitation with anti-NLRP3 antibody revealed that NLRP3 associated with MAVS in BMDMs treated with LPS plus nigericin, and its binding remained in the absence of MARCH5. In addition, ubiquitination-defective NLRP3 K324R and K430R mutants remained bound to MAVS (Appendix Fig S1C). However, depletion of MAVS by siRNA reduced the interaction of MARCH5 with NLRP3, which furthermore disrupted the interaction of ASC with NLRP3 (Appendix Fig S1D). Thus, the data suggest that NLRP3 interaction with MAVS is preceded and the stabilized NLRP3 at the mitochondrial outer membrane is subsequently

ubiquitinated by MARCH5.”

2nd Revision - Editorial Decision 23rd Jul 2023

Dear Hyeseong,

Thanks for sending us your revised manuscript. I have now looked at everything and all looks good! I am therefore very pleased to accept the manuscript for publication here.

Congratulation on a nice study!

with best wishes

Karin

Karin Dumstrei, PhD
Senior Editor
The EMBO Journal

Please note that it is EMBO Journal policy for the transcript of the editorial process (containing referee reports and your response letter) to be published as an online supplement to each paper. If you do NOT want this, you will need to inform the Editorial Office via email immediately. More information is available here:
https://www.embopress.org/page/journal/14602075/authorguide#transparentprocess

Your manuscript will be processed for publication in the journal by EMBO Press. Manuscripts in the PDF and electronic editions of The EMBO Journal will be copy edited, and you will be provided with page proofs prior to publication. Please note that supplementary information is not included in the proofs.

You will be contacted by Wiley Author Services to complete licensing and payment information. The required 'Page Charges Authorization Form' is available here: https://www.embopress.org/pb-assets/embo-site/tej_apc.pdf - please download and complete the form and return to embopressproduction@wiley.com

EMBO Press participates in many Publish and Read agreements that allow authors to publish Open Access with reduced/no publication charges. Check your eligibility: https://authorservices.wiley.com/author-resources/Journal-Authors/open-access/affiliation-policies-payments/index.html

Should you be planning a Press Release on your article, please get in contact with embojournal@wiley.com as early as possible, in order to coordinate publication and release dates.

If you have any questions, please do not hesitate to call or email the Editorial Office. Thank you for your contribution to The EMBO Journal.

EMBO Press Author Checklist

Corresponding Author Name: Hyeseong Cho, Jong-Soo Lee	
Journal Submitted to: The EMBO Journal	
Manuscript Number: EMBOJ-2023-113481R	

USEFUL LINKS FOR COMPLETING THIS FORM
The EMBO Journal - Author Guidelines
EMBO Reports - Author Guidelines
Molecular Systems Biology - Author Guidelines
EMBO Molecular Medicine - Author Guidelines

Reporting Checklist for Life Science Articles (updated January
This checklist is adapted from Materials Design Analysis Reporting (MDAR) Checklist for Authors. MDAR establishes a minimum set of requirements in transparent reporting in the life sciences (see Statement of Task: 10.31222/osf.io/9sm4x). Please follow the journal's guidelines in preparing your manuscript.
Please note that a copy of this checklist will be published alongside your article.

Abridged guidelines for figures
1. Data
The data shown in figures should satisfy the following conditions:
- the data were obtained and processed according to the field's best practice and are presented to reflect the results of the experiments in an accurate and unbiased manner.
- ideally, figure panels should include only measurements that are directly comparable to each other and obtained with the same assay.
- plots include clearly labeled error bars for independent experiments and sample sizes. Unless justified, error bars should not be shown for technical
- if n<5, the individual data points from each experiment should be plotted. Any statistical test employed should be justified.
- Source Data should be included to report the data underlying figures according to the guidelines set out in the authorship guidelines on Data

2. Captions
Each figure caption should contain the following information, for each panel where they are relevant:
- a specification of the experimental system investigated (eg cell line, species name).
- the assay(s) and method(s) used to carry out the reported observations and measurements.
- an explicit mention of the biological and chemical entity(ies) that are being measured.
- an explicit mention of the biological and chemical entity(ies) that are altered/varied/perturbed in a controlled manner.
- the exact sample size (n) for each experimental group/condition, given as a number, not a range;
- a description of the sample collection allowing the reader to understand whether the samples represent technical or biological replicates (including how many animals, litters, cultures, etc.).
- a statement of how many times the experiment shown was independently replicated in the laboratory.
- definitions of statistical methods and measures:
  - common tests, such as t-test (please specify whether paired vs. unpaired), simple χ2 tests, Wilcoxon and Mann-Whitney tests, can be unambiguously identified by name only, but more complex techniques should be described in the methods section;
  - are tests one-sided or two-sided?
  - are there adjustments for multiple comparisons?
  - exact statistical test results, e.g., P values = x but not P values < x;
  - definition of 'center values' as median or average;
  - definition of error bars as s.d. or s.e.m.

Please complete ALL of the questions below.
Select "Not Applicable" only when the requested information is not relevant for your study.

Materials

Newly Created Materials	Information included in the manuscript?	In which section is the information available? (Reagents and Tools Table, Materials and Methods, Figures, Data Availability Section)
New materials and reagents need to be available; do any restrictions apply?	Yes	Materials and Methods

Antibodies	Information included in the manuscript?	In which section is the information available? (Reagents and Tools Table, Materials and Methods, Figures, Data Availability Section)
For **antibodies** provide the following information:<br>- Commercial antibodies: RRID (if possible) or supplier name, catalogue number and or/clone number<br>- Non-commercial: RRID or citation	Yes	Materials and Methods, Reagents and Tools Table

DNA and RNA sequences	Information included in the manuscript?	In which section is the information available? (Reagents and Tools Table, Materials and Methods, Figures, Data Availability Section)
**Short novel DNA or RNA including primers, probes**: provide the sequences.	Yes	Materials and Methods, Reagents and Tools Table

Cell materials	Information included in the manuscript?	In which section is the information available? (Reagents and Tools Table, Materials and Methods, Figures, Data Availability Section)
**Cell lines:** Provide species information, strain. Provide accession number in repository **OR** supplier name, catalog number, clone number, and/**OR** RRID.	Yes	Materials and Methods, Reagents and Tools Table
**Primary cultures:** Provide species, strain, sex of origin, genetic modification status.	Yes	Materials and Methods
Report if the cell lines were recently **authenticated** (e.g., by STR profiling) and tested for mycoplasma contamination.	Not Applicable	

Experimental animals	Information included in the manuscript?	In which section is the information available? (Reagents and Tools Table, Materials and Methods, Figures, Data Availability Section)
**Laboratory animals or Model organisms:** Provide species, strain, sex, age, genetic modification status. Provide accession number in repository **OR** supplier name, catalog number, clone number, **OR** RRID.	Yes	Materials and Methods,
**Animal observed in or captured from the field:** Provide species, sex, and age where possible.	Yes	Materials and Methods,
Please detail **housing and husbandry conditions**.	Yes	Materials and Methods

Plants and microbes	Information included in the manuscript?	In which section is the information available? (Reagents and Tools Table, Materials and Methods, Figures, Data Availability Section)
**Plants:** provide species and strain, ecotype and cultivar where relevant, unique accession number if available, and source (including location for collected wild specimens).	Not Applicable	
**Microbes:** provide species and strain, unique accession number if available, and source.	Yes	Materials and Methods, Reagents and Tools Table

Human research participants	Information included in the manuscript?	In which section is the information available? (Reagents and Tools Table, Materials and Methods, Figures, Data Availability Section)
If collected and within the bounds of privacy constraints report on age, sex and gender or ethnicity for all study participants.	Not Applicable	

Core facilities	Information included in the manuscript?	In which section is the information available? (Reagents and Tools Table, Materials and Methods, Figures, Data Availability Section)
If your work benefited from core facilities, was their service mentioned in the acknowledgments section?	Not Applicable	

Design

Study protocol	Information included in the manuscript?	In which section is the information available? (Reagents and Tools Table, Materials and Methods, Figures, Data Availability Section)
If study protocol has been **pre-registered, provide DOI in the manuscript**. For clinical trials, provide the trial registration number **OR** cite DOI.	Not Applicable	
Report the **clinical trial registration number** (at ClinicalTrials.gov or equivalent), where applicable.	Not Applicable	

Laboratory protocol	Information included in the manuscript?	In which section is the information available? (Reagents and Tools Table, Materials and Methods, Figures, Data Availability Section)
Provide DOI OR other citation details if **external detailed step-by-step protocols** are available.	Not Applicable	

Experimental study design and statistics	Information included in the manuscript?	In which section is the information available? (Reagents and Tools Table, Materials and Methods, Figures, Data Availability Section)
Include a statement about **sample size** estimate even if no statistical methods were used.	Not Applicable	
Were any steps taken to minimize the effects of subjective bias when allocating animals/samples to treatment (e.g. **randomization procedure**)? If yes, have they been described?	Not Applicable	
Include a statement about **blinding** even if no blinding was done.	Not Applicable	
Describe **inclusion/exclusion criteria** if samples or animals were excluded from the analysis. Were the criteria pre-established?<br><br>If sample or data points were omitted from analysis, report if this was due to attrition or intentional exclusion and provide justification.	Not Applicable	
For every figure, are **statistical tests** justified as appropriate? Do the data meet the assumptions of the tests (e.g., normal distribution)? Describe any methods used to assess it. Is there an estimate of variation within each group of data? Is the variance similar between the groups that are being statistically compared?	Yes	Figures, Materials and Methods

Sample definition and in-laboratory replication	Information included in the manuscript?	In which section is the information available? (Reagents and Tools Table, Materials and Methods, Figures, Data Availability Section)
In the figure legends: state number of times the experiment was **replicated** in laboratory.	Yes	Figures
In the figure legends: define whether data describe **technical or biological replicates**.	Yes	Figures

Ethics

Ethics	Information included in the manuscript?	In which section is the information available? (Reagents and Tools Table, Materials and Methods, Figures, Data Availability Section)
Studies involving **human participants**: State details of **authority granting ethics approval** (IRB or equivalent committee(s), provide reference number for approval.	Not Applicable	
Studies involving **human participants**: Include a statement confirming that **informed consent** was obtained from all subjects and that the experiments conformed to the principles set out in the WMA Declaration of Helsinki and the Department of Health and Human Services Belmont Report.	Not Applicable	
Studies involving **human participants:** For publication of **patient photos**, include a statement confirming that consent to publish was obtained.	Not Applicable	
Studies involving experimental **animals**: State details of **authority granting ethics approval** (IRB or equivalent committee(s), provide reference number for approval. Include a statement of compliance with ethical regulations.	Yes	Meterials and Methods
Studies involving **specimen and field samples:** State if relevant **permits** obtained, provide details of authority approving study; if none were required, explain why.	Not Applicable	

Dual Use Research of Concern (DURC)	Information included in the manuscript?	In which section is the information available? (Reagents and Tools Table, Materials and Methods, Figures, Data Availability Section)
Could your study fall under dual use research restrictions? Please check biosecurity documents and list of **select agents and toxins** (CDC): https://www.selectagents.gov/sat/list.htm	Not Applicable	
If you used a select agent, is the security level of the lab appropriate and reported in the manuscript?	Not Applicable	
If a study is subject to dual use research of concern regulations, is the name of the **authority granting approval and reference number** for the regulatory approval provided in the manuscript?	Not Applicable	

Reporting

The MDAR framework recommends adoption of discipline-specific guidelines, established and endorsed through community initiatives. Journals have their own policy about requiring specific guidelines and recommendations to complement MDAR.

Adherence to community standards	Information included in the manuscript?	In which section is the information available? (Reagents and Tools Table, Materials and Methods, Figures, Data Availability Section)
State if relevant guidelines or checklists (e.g., **ICMJE, MIBBI, ARRIVE, PRISMA**) have been followed or provided.	Not Applicable	
For **tumor marker prognostic studies,** we recommend that you follow the **REMARK** reporting guidelines (see link list at top right). See author guidelines, under 'Reporting Guidelines'. Please confirm you have followed these guidelines.	Not Applicable	
For **phase II and III randomized controlled trials**, please refer to the **CONSORT** flow diagram (see link list at top right) and submit the CONSORT checklist (see link list at top right) with your submission. See author guidelines, under 'Reporting Guidelines'. Please confirm you have submitted this list.	Not Applicable	

Data Availability

Data availability	Information included in the manuscript?	In which section is the information available? (Reagents and Tools Table, Materials and Methods, Figures, Data Availability Section)
Have **primary datasets** been deposited according to the journal's guidelines (see 'Data Deposition' section) and the respective accession numbers provided in the Data Availability Section?	Not Applicable	
Were **human clinical and genomic datasets** deposited in a public access-controlled repository in accordance to ethical obligations to the patients and to the applicable consent agreement?	Not Applicable	
Are **computational models** that are central and integral to a study available without restrictions in a machine-readable form? Were the relevant accession numbers or links provided?	Not Applicable	
If publicly available data were reused, provide the respective **data citations in the reference list**.	Not Applicable	
