# Supplementary material for: MARCH5‐dependent NLRP3 ubiquitination is required for mitochondrial NLRP3‐NEK7 complex formation and NLRP3 inflammasome activation
Source: EMBO J. 2023 Aug 14;42(19):e113481. doi: 10.15252/embj.2023113481 (PMC10548170; doi:10.15252/embj.2023113481)

Figure EV1

B

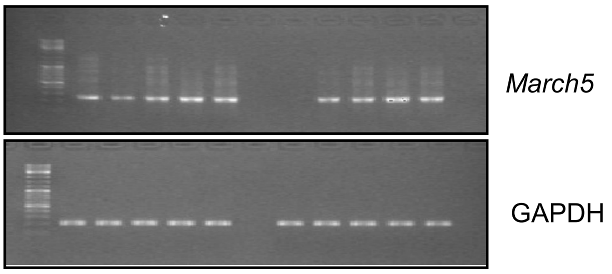

C

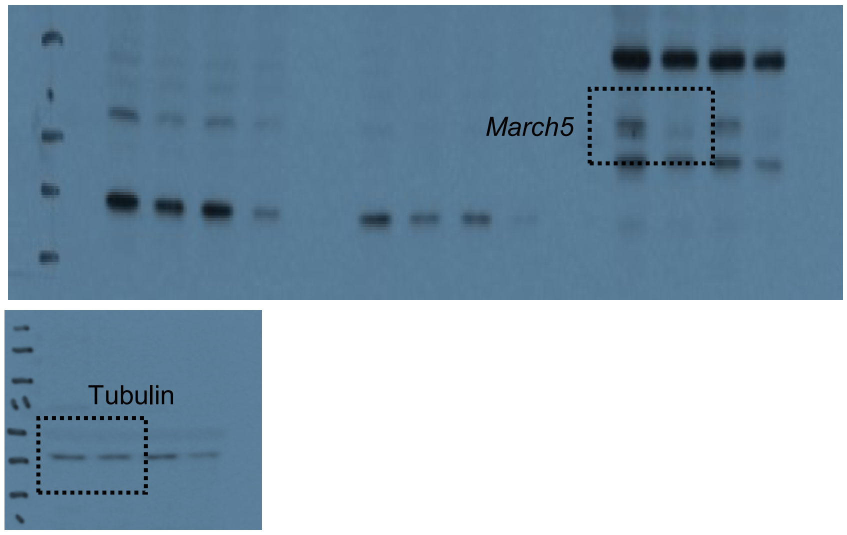

Supplement: Supplementary file 3 — Source Data for Expanded View and Appendix [file EMBJ-42-e113481-s002.zip › EV and Appendix Figure_SourceData/EMBOJ-2023-113481R-Figure EV1 Source Data.zip!/EMBOJ-2023-113481R-Figure EV1/Figure EV1.pdf]
