# Supplementary material for: MARCH5‐dependent NLRP3 ubiquitination is required for mitochondrial NLRP3‐NEK7 complex formation and NLRP3 inflammasome activation
Source: EMBO J. 2023 Aug 14;42(19):e113481. doi: 10.15252/embj.2023113481 (PMC10548170; doi:10.15252/embj.2023113481)
Supplement: Supplementary file 3 — Source Data for Expanded View and Appendix [file EMBJ-42-e113481-s002.zip › EV and Appendix Figure_SourceData/EMBOJ-2023-113481R-Figure EV4 Source Data.zip!/EMBOJ-2023-113481R-Figure EV4/EV4 western blot.pdf]

# Figure EV4

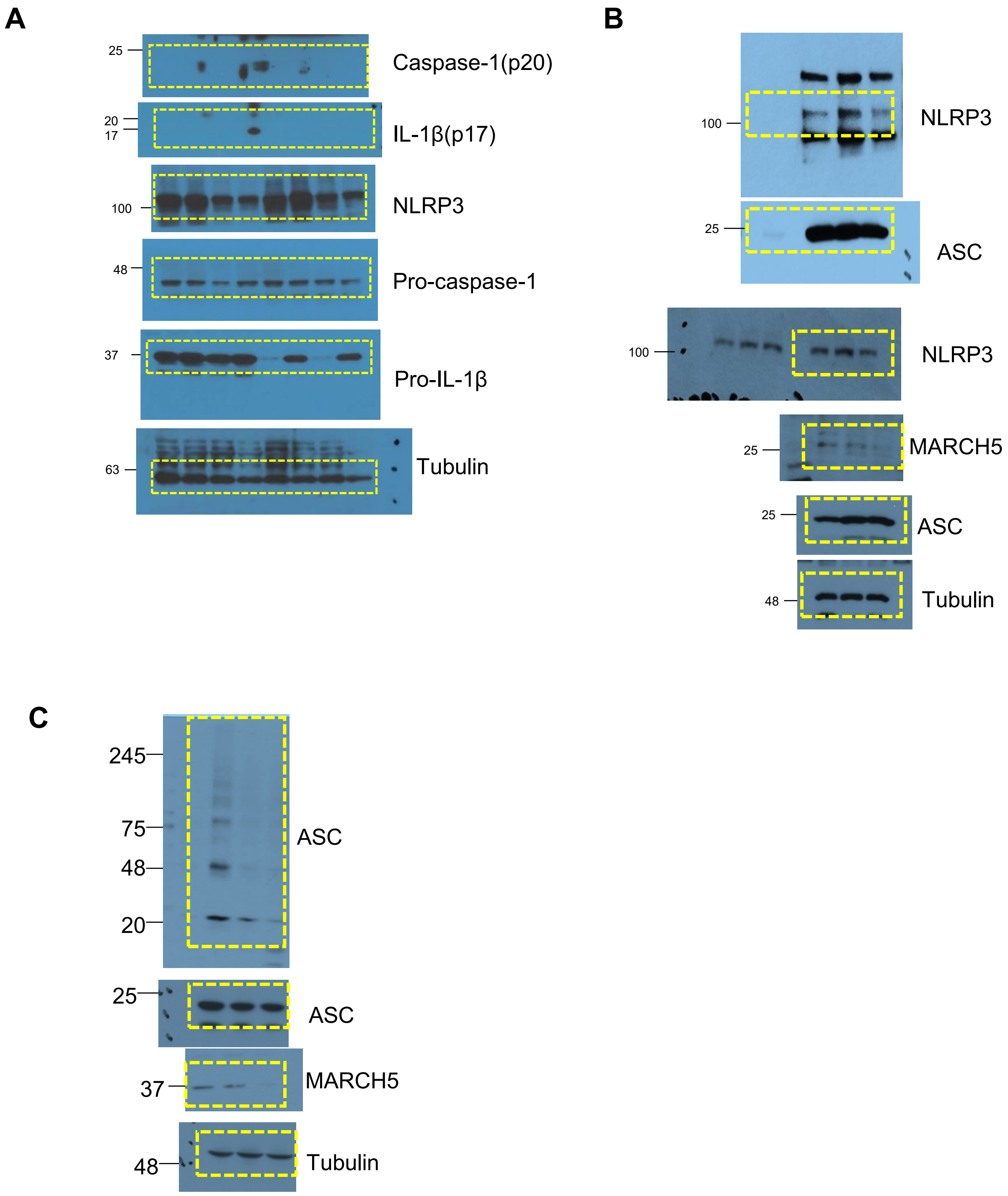
A
25
Caspase-1(p20)
20
17
IL-1β(p17)
100
NLRP3
48
Pro-caspase-1
37
Pro-IL-1β
63
Tubulin
B
100
NLRP3
25
ASC
100
NLRP3
25
MARCH5
25
ASC
48
Tubulin
C
245
75
48
20
ASC
25
ASC
37
MARCH5
48
Tubulin
