# Supplementary material for: MARCH5‐dependent NLRP3 ubiquitination is required for mitochondrial NLRP3‐NEK7 complex formation and NLRP3 inflammasome activation
Source: EMBO J. 2023 Aug 14;42(19):e113481. doi: 10.15252/embj.2023113481 (PMC10548170; doi:10.15252/embj.2023113481)
Supplement: Supplementary file 3 — Source Data for Expanded View and Appendix [file EMBJ-42-e113481-s002.zip › EV and Appendix Figure_SourceData/EMBOJ-2023-113481R-Figure EV5 Source Data.zip!/EMBOJ-2023-113481R-Figure EV5/EV5 western blot.pdf]

Figure EV5

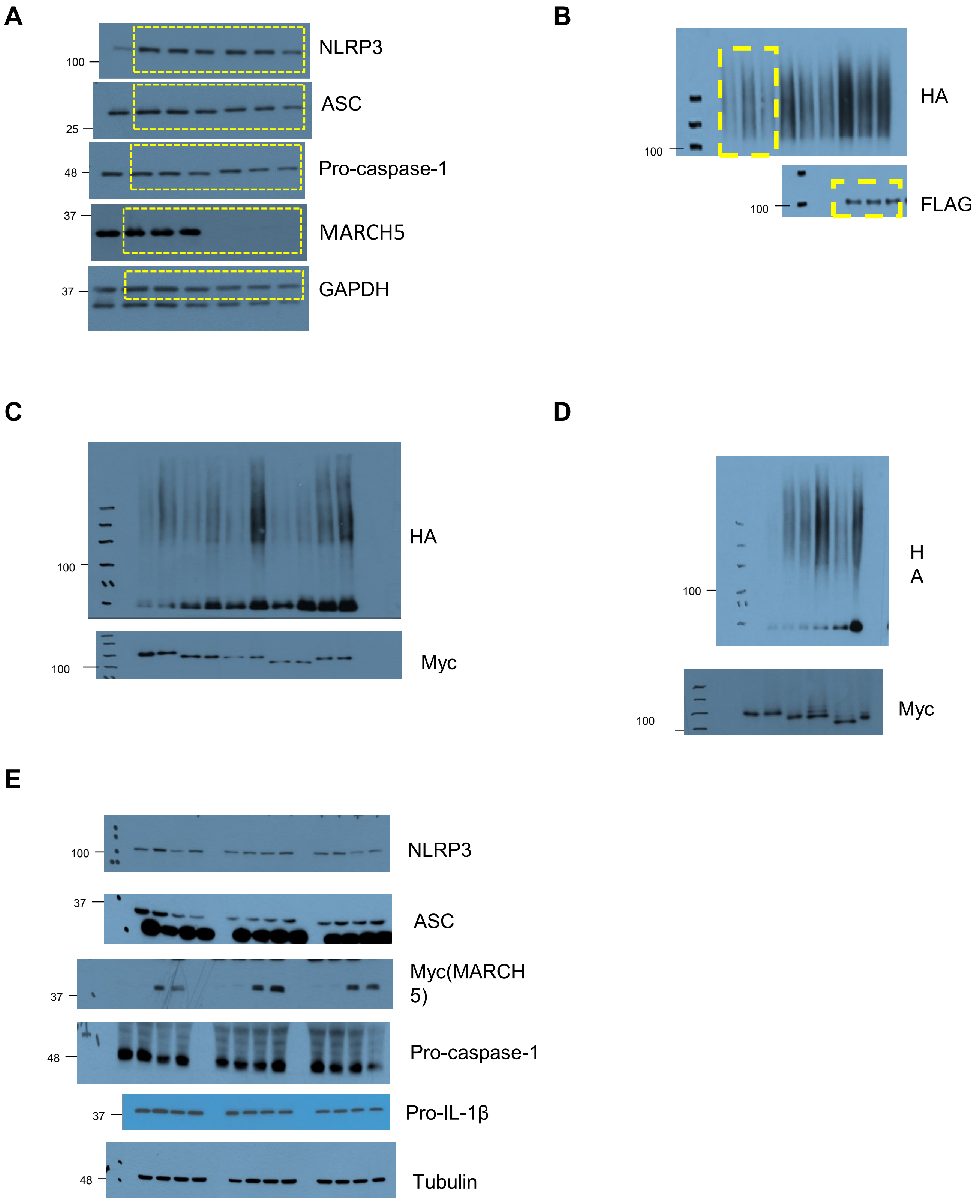
A
100
NLRP3
25
ASC
48
Pro-caspase-1
37
MARCH5
37
GAPDH
B
100
HA
100
FLAG
C
100
HA
100
Myc
D
100
H
A
100
Myc
E
100
NLRP3
37
ASC
37
Myc(MARCH
5)
48
Pro-caspase-1
37
Pro-IL-1β
48
Tubulin
